# Dual antiplatelet therapy for ischemic stroke with intracranial arterial stenosis: a systematic review and meta-analysis

**DOI:** 10.3389/fneur.2024.1411669

**Published:** 2024-06-10

**Authors:** Haifeng Shao, Song He, Ping Ni, Danni Zheng, Nengwei Yu, Qiao Chen, Xinyi Leng, Yan Lin, Suping Li, Jie Yang, Xia Wang

**Affiliations:** ^1^School of Medical and Life Sciences, Chengdu University of Traditional Chinese Medicine, Chengdu, China; ^2^Department of Neurology, The First Affiliated Hospital of Chengdu Medical College, Chengdu, China; ^3^Department of Neurology, Sichuan Provincial People’s Hospital, University of Electronic Science and Technology of China, Chengdu, China; ^4^The George Institute for Global Health, University of New South Wales, Sydney, NSW, Australia; ^5^Department of Medicine & Therapeutics, The Chinese University of Hong Kong, Shatin, Hong Kong SAR, China; ^6^Faculty of Medicine, The George Institute for Global Health, University of New South Wales, Sydney, NSW, Australia

**Keywords:** ischemic stroke, intracranial artery stenosis, dual antiplatelet therapy, bleeding event, systematic review, meta-analysis

## Abstract

**Background:**

The safety and efficacy of dual antiplatelet therapy (DAPT) in ischemic stroke patients with intracranial artery stenosis (ICAS) remain contentious.

**Aims:**

This study evaluates DAPT’s effectiveness and safety for these patients.

**Methods:**

This review was reported following PRISMA 2020 guidelines. A comprehensive search was conducted in PubMed, Embase, Cochrane Library, ClinicalTrials.gov, CNKI, WanFang, VIP, and SinoMed up to June 20, 2023, for randomized controlled trials comparing efficacy and safety of DAPT against single antiplatelet therapy (SAPT) in ischemic stroke patients with ICAS. The primary outcome was a composite of ischemic and bleeding events. Secondary outcomes included stroke (cerebral infarction and hemorrhage), ischemic events, and cerebral infarction. Safety outcomes assessed were bleeding events, cerebral hemorrhage, and mortality. Risk ratios (RRs) with 95% confidence intervals (CIs) were synthesized using Review Manager 5.4.

**Results:**

Analysis of 21 randomized controlled trials involving 3,591 patients revealed that DAPT significantly lowered the rate of ischemic and bleeding events (RR = 0.52; 95% CI: 0.46–0.59, *p* < 0.001) and recurrent stroke (RR = 0.37; 95% CI: 0.30–0.44, *p* < 0.001) compared to SAPT. There was no significant increase in bleeding events (RR = 1.34; 95% CI: 0.97–1.85, *p* = 0.07) or cerebral hemorrhage (RR = 0.47; 95% CI: 0.17–1.31, *p* = 0.15).

**Conclusion:**

DAPT proveed to be effective and safe for ischemic stroke patients with ICAS and significantly reduced stroke and the composite endpoint of ischemic and bleeding events without elevating bleeding risks.

## Introduction

Ischemic stroke is the necrosis of brain tissue caused by cerebral artery stenosis or occlusion and insufficient cerebral blood supply (1–3). Patients develop clinical symptoms that are difficult to resolve in a short time, including unilateral limb weakness or numbness, unclear speech, blurred vision, nausea and vomiting, disturbance of consciousness and so on (1). Ischemic stroke is the leading cause of disability and death worldwide and has a high recurrence (1, 4). Intracranial artery stenosis (ICAS) significantly increases the risk of ischemic stroke recurrence and worsens outcomes (5–9). Antiplatelet medications are crucial for lowering post-stroke thrombosis risk (10, 11). Although single antiplatelet therapy (SAPT) is commonly used, it often fails to reduce stroke recurrence in patients with ICAS adequately. Dual antiplatelet therapy (DAPT), combining agents with different mechanisms, has shown promise in enhancing ischemic event prevention (12–14). Despite some small-scale studies suggesting DAPT’s efficacy and safety in ICAS patients (15–20), evidence remains sparse and debated. This systematic review aims to clarify DAPT’s effectiveness and safety in treating new non-cardiac ischemic stroke patients with ICAS.

## Methods

### Search strategy

The systematic review and meta-analysis were prepared following the Preferred Reporting Items for Systematic Reviews and Meta-Analyses (PRISMA) 2020 statement (21). The databases PubMed, Embase, Cochrane Library, Clinicaltrials.gov, CNKI, WanFang, VIP, and SinoMed were searched from inception through June 20, 2023. We used keywords associated with ischemic stroke (e.g., “ischemic stroke,” “cerebral infarction,” “stroke,” “cerebrovascular disease”), intracranial artery stenosis (e.g., “intracranial artery stenosis,” “cerebral atherosclerosis,” “intracranial atherosclerotic stenosis”) and antiplatelet drugs (e.g., “antiplatelet drugs,” “aspirin,” “clopidogrel,” “ticagrelor,” “cilostazol,” “dipyridamole”). The search methodologies are detailed in the Supplemental material. We also scrutinized the reference lists of relevant articles to identify additional studies that could meet our eligibility criteria.

### Selection criteria

Our inclusion criteria were structured following the PICOS framework (22): (1) randomized controlled trials (RCTs) that enrolled patients with ischemic stroke patients with ICAS [The existence of ICAS was confirmed by CTA, MRA, DSA or other imaging methods. The classification of ischemic stroke in all trials followed the standard of TOAST classification (23, 24) (Supplementary Table S1)] and included patients aged over 18; (2) studies that compared DAPT with SAPT, using agents like aspirin, clopidogrel, ticagrelor, cilostazol, dipyridamole etc.; and (3) studies that reported any of the following outcomes including composite ischemic and bleeding events, stroke, ischemic events (including TIA and myocardial infarction), the National Institute of Heath Stroke Scale (NIHSS), and modified Rankin Scale (mRS) scores, various bleeding events [bleeding was defined as any bleeding event that met the BARC2, 3 or 5 criteria (25)] and death. We excluded studies with non ICAS population, subgroup analysis from large-scale RCTs, comparisons of DAPT with non-antiplatelet or anticoagulant therapies, those lacking pertinent data, non-RCTs, observational studies, reviews, meta-analyses, and trials scoring under 3 on the modified Jadad scale (26) for quality.

### Selection of studies

Two review authors (HS and SH) independently screened the title, abstract, and full text of the articles, excluding irrelevant studies, and independently determined included trials based on inclusion and exclusion criteria. Any disagreements were resolved by a third author (SL).

### Article quality assessment

The modified Jadad scale was used to assess the quality of the 21 included randomized controlled trials. Two reviewers (HS and PN) evaluated the quality of the included trials according to the modified Jadad scale, and a third reviewer (SL) resolved any disagreements.

### Data extraction and management

Two reviewers (HS and SH) independently extracted the following data from the eligible studies: first author’s name, publication time, study design, sample size, type and dose of antiplatelet regimes, duration, location of ICAS, follow-up time, and the frequency of outcome events. The same two reviewers cross-checked the above data and discussed the acceptability. Any disagreements were decided by a third reviewer (JY).

### Statistical analysis

The effect of DAPT vs. SAPT was calculated using relative risk (RR) and its 95% confidence interval (CI) or mean difference (MD) and its 95% CI. Estimation of publication bias was performed using a funnel plot. I^2^ value was used to assess heterogeneity between studies. A fixed effect model was used if I^2^ ≤ 50%, and random effect model was used If I^2^ > 50%. *p*-values <0.05 were considered statistically significant. The statistical analyses were conducted by Review Manager 5.4.

### Outcome measures

Although DAPT proved superior to SAPT in preventing recurrent ischemic strokes, the risk of hemorrhage events was significantly increased (27). Therefore, we defined primary outcome as a composite of ischemic and bleeding events. Secondary outcomes were stroke (cerebral infarction and hemorrhage), ischemic events, and cerebral infarction. The safety outcomes assessed were bleeding events [defined as any bleeding event that met the BARC2, 3 or 5 criteria (25)], cerebral hemorrhage, and mortality.

## Results

### Study selection

**Figure 1:**
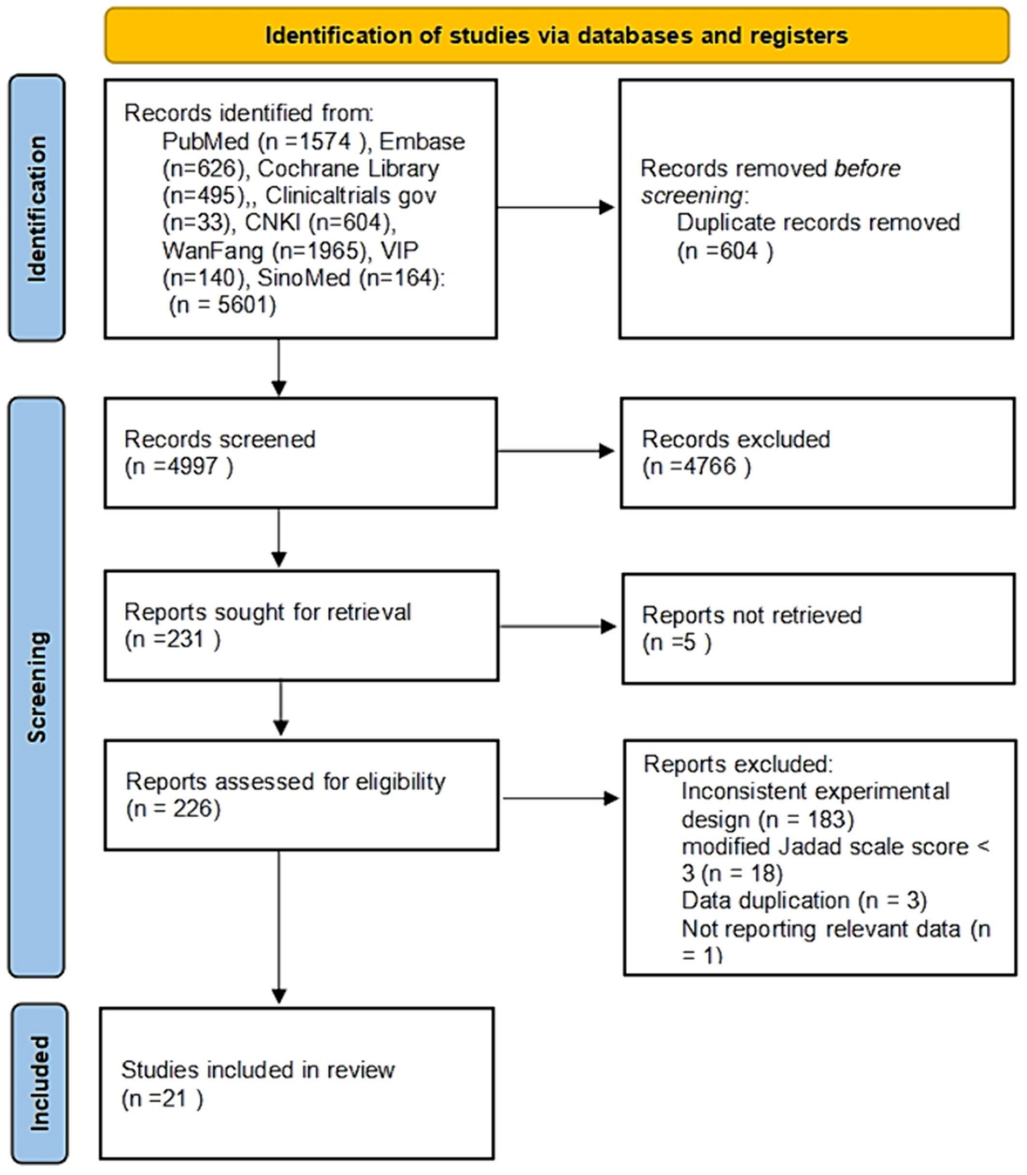
Study selection flowchart.

After a detailed search of the above databases, 5,601 records were identified. 4,997 were left after removing the duplicate articles. After carefully reviewing the titles and abstracts. We found 226 articles relevant to our review, of which 205 were excluded after the full-text review. Among them, 183 articles did not meet the inclusion criteria, 18 scored less than 3 with the modified Jadad scale, 1 lacked detailed data, and the rest were excluded because of repeated data. Finally, 21 randomized controlled trials were included in this review (Figure 1).

### Study characteristics

A total of 3,591 patients were included, including 1951 and 1,640 patients in the DAPT and SAPT groups. In the included studies, DAPTs were mainly aspirin combined with clopidogrel, aspirin combined with cilostazol or dipyridamole, or SAPT with aspirin or clopidogrel. As for the drug dose, the standard doses were 100 mg of aspirin and 75 mg of clopidogrel, daily. Some treatments started with an initial dose of 300 mg daily before decreasing to the standard dose. DAPT duration ranged from 7 days to 2 years, with most treatments lasting around 2 months. The stenotic arteries in the included studies were mainly the middle cerebral artery of the anterior circulation, and only one was the posterior circulation artery. The degree of stenosis of most blood vessels is more than 50%, some of which are not clearly stated. The characteristics of the included studies are listed in Table 1.

**Table 1:** Characteristics of the included studies.

Study	Study type	Race	Sample size	DAPT	SAPT	DAPT time	Age(mean ± SD years)	Female (%)	Follow-up time	Outcome
Wang (28)	RCT	Asia	100	Aspirin + clopidogrel	Aspirin	2 months	57.5 ± 3.3/57.5 ± 3.2	47.0	2 months	(3)
Liu et al. (29)	RCT	Asia	60	Aspirin + clopidogrel	Aspirin	3 months	NA	33.3	1 year	(2)(3)(6)
Hao et al. (30)	RCT	Asia	120	Aspirin + clopidogrel	Aspirin / clopidogrel	7 days	62.3 ± 6.6/61.2 ± 7.3	28.3	3 months	(2)(3)(6)
Ma (31)	RCT	Asia	58	Aspirin + clopidogrel	Aspirin	3 months	59.2 ± 2.2/58.4 ± 2.1	43.1	1 year	(2)(3)
Jin (32)	RCT	Asia	84	Aspirin + clopidogrel	Aspirin	NA	68.2 ± 1.7/67.8 ± 2.1	44.0	NA	(3)
Fan (33)	RCT	Asia	118	Aspirin + clopidogrel	Aspirin	2 months	52.8 ± 7.1/53.2 ± 6.9	46.6	2 months	(3)
Ge et al. (16)	RCT	Asia	138	Aspirin + clopidogrel	Aspirin / clopidogrel	3 months	63.7 ± 10.1/65.1 ± 8.9	40.0	6 months	(2)(3)(5)(6)
Wang (34)	RCT	Asia	76	Aspirin + clopidogrel	Aspirin	2 months	57.9 ± 6.8/57.9 ± 6.8	46.0	1 year	(2)(3)
Liu (35)	RCT	Asia	68	Aspirin + clopidogrel	Aspirin	2 months	65.4 ± 3.7/65.1 ± 3.2	45.6	NA	(3)(6)
Lin and Ou (36)	RCT	Asia	168	Aspirin + clopidogrel	Aspirin	2 months	61.8 ± 5.9/61.5 ± 4.8	42.9	1 year	(2)(3)
Cheng (37)	RCT	Asia	86	Aspirin + clopidogrel	Aspirin	NA	55.2 ± 4.8/56.0 ± 4.3	47.7	1 year	(2)(3)
Tai (38)	RCT	Asia	132	Aspirin + clopidogrel	Aspirin	2 months	56.6 ± 6.8/55.1 ± 7.2	43.2	1 year	(2)
Liang (39)	RCT	Asia	114	Aspirin + clopidogrel	Aspirin	2 months	54.2 ± 8.3/55.2 ± 8.7	47.4	1 year	(2)
Li (40)	RCT	Asia	56	Aspirin + clopidogrel	Aspirin	2 months	NA	35.7	1 year	(2)
Li (41)	RCT	Asia	62	Aspirin + clopidogrel	Aspirin	2 months	NA	40.3	1 year	(2)(3)
Mao (42)	RCT	Asia	150	Aspirin + clopidogrel	clopidogrel	2 months	52.9 ± 10.3/52.9 ± 10.3	38.7	NA	(3)
Qu et al. (19)	RCT	Asia	53	Aspirin + clopidogrel	clopidogrel	3 months	64.5 ± 6.9/63.3 ± 7.1	39.6	1 year	(2)(3)
Wang et al. (43)	RCT	Asia	1,126	Aspirin + dipyridamole	Aspirin	6 months	61.6 ± 7.3/60.5 ± 8.3	38.2	5 years	(1)(6)(7)
Jiang et al. (20)	RCT	Asia	89	Aspirin + clopidogrel	Aspirin	1 month	64.5 ± 0.0/66.5 ± 0.0	32.6	DAPT:20.7 ± 8.1 months SAPT:19,2 ± 11.4 months	(1)(7)
Wang et al. (17)	RCT	Asia	570	Aspirin + clopidogrel	Aspirin	1 month	69.2 ± 10.1/70.1 ± 10.4	45.1	6 months	(1)(6)
Uchiyama et al. (18)	RCT	Asia	163	Aspirin + cilostazol	Aspirin	2 years	68.3 ± 0.0/68.3 ± 0.0	34.4	2 years	(1)(7)

RCT, randomized controlled trial; DAPT, dual antiplatelet therapy; SAPT, single antiplatelet therapy; SD, standard deviation. (1) Ischemic events; (2) cerebral infarction; (3) NIHSS; (4) mRS; (5) death; (6) bleeding events; (7) cerebral hemorrhage.

### Study quality assessment

Scores of the modified Jadad scale were distributed as follows: 10 studies scored 3 points, 10 studies scored 4 points, and 1 study achieved 6 points, as detailed in Supplementary Table S2. This scoring indicates that the overall quality of the included trials was moderate to acceptable.

### Publication bias

**Figure 2:**
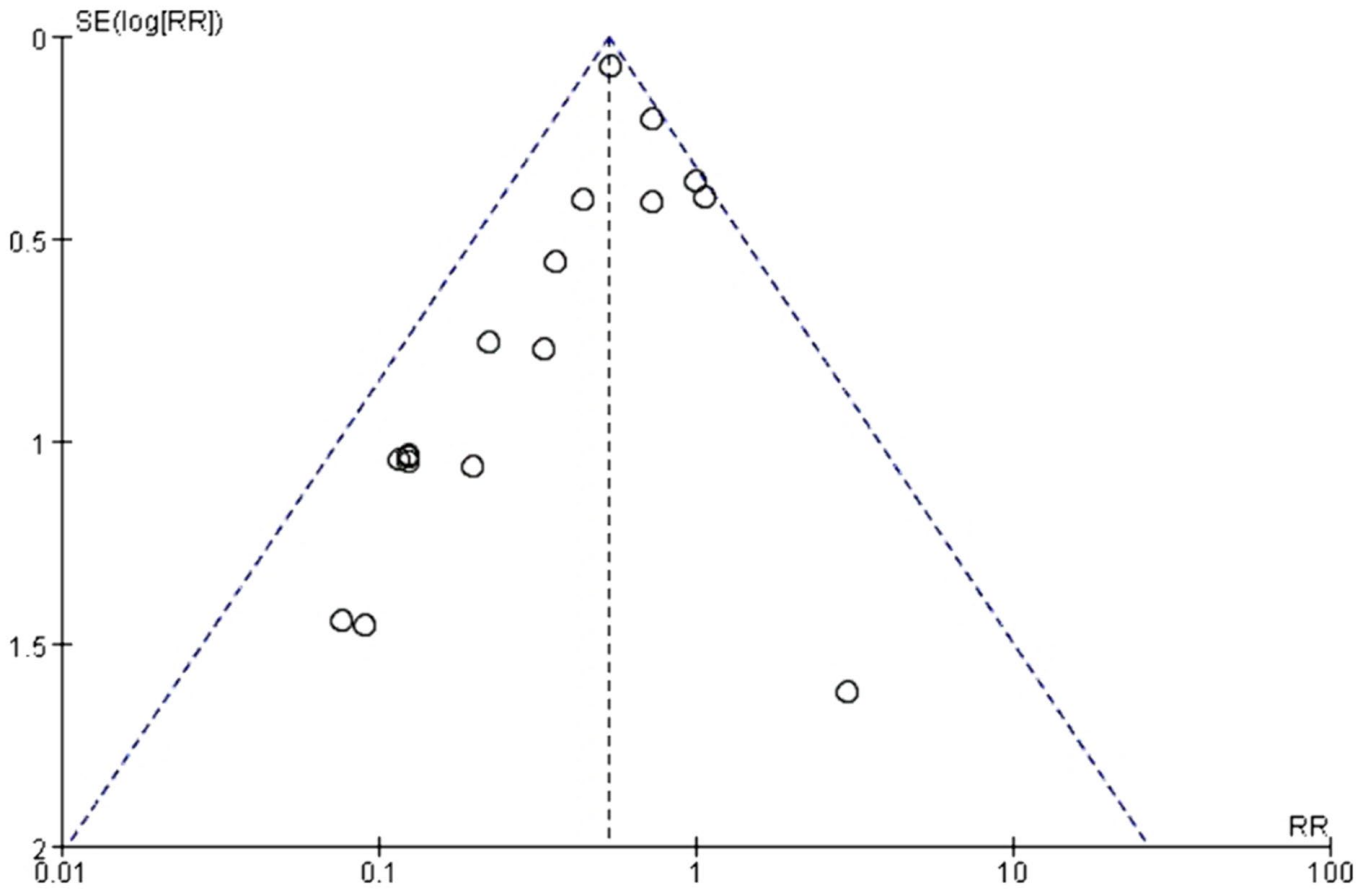
Funnel plot for the included studies.

The symmetry of the plot indicates an absence of significant publication bias in this review, as illustrated in Figure 2.

## Outcomes and meta-analysis

**Figure 3:**
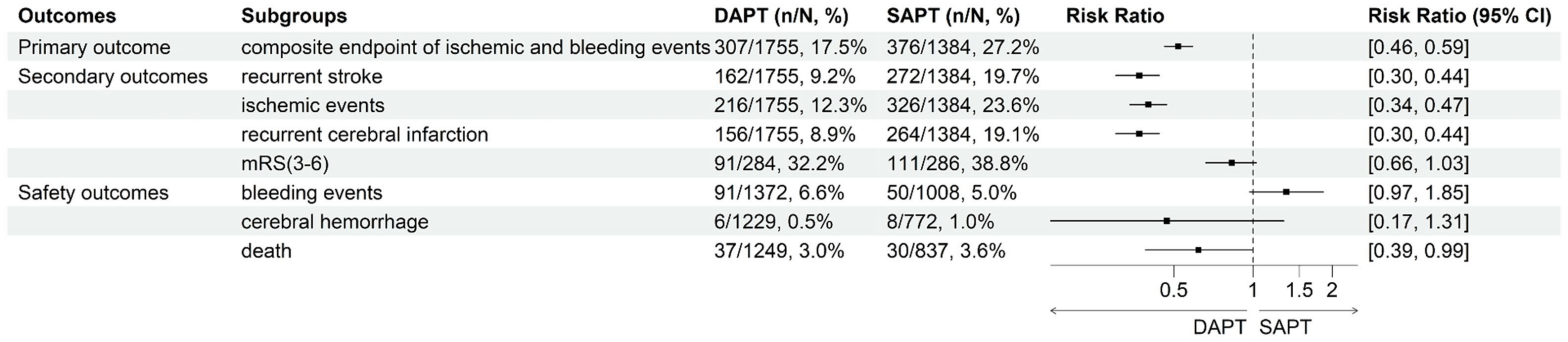
Forest plot of overall meta-analysis results.

The overall meta-analysis result of primary, secondary, and safety outcomes is shown in Figure 3.

### Primary outcome – the composite of ischemic and bleeding events

Seventeen trials provided data on the events (15–20, 29, 31, 34–41, 43). In these studies, composite endpoint events were reported in 307 participants (17.5%) receiving DAPT, compared to 376 participants (27.2%) in the SAPT group. Meta-analysis results showed a significant reduction in composite endpoint events in the DAPT group [RR = 0.52, 95% CI: 0.46–0.59 *p* < 0.001; I^2^ = 34%; Supplementary Figure S1].

### Secondary outcomes – recurrent stroke occurrence, ischemic events, recurrent cerebral infarction, NIHSS, and mRS(3–6)

Data on recurrent stroke occurrence were reported in seventeen trials (15–20, 29, 31, 34–41, 43). Among these, recurrent stroke was observed in 162 participants (9.2%) within the DAPT group versus 272 participants (19.7%) in the SAPT group. The analysis indicated that the risk of recurrent stroke was significantly lower in those treated with DAPT [RR = 0.37; 95%CI: 0.30–0.44 *p* < 0.001; I^2^ = 0%; Supplementary Figure S2].

Seventeen trials provided data on ischemic events (15–20, 29, 31, 34–41, 43). These events were reported in 216 participants (12.3%) receiving DAPT, compared to 326 participants (23.6%) in the SAPT group. Analysis showed that DAPT significantly reduced the risk of ischemic events [RR = 0.40; 95% CI: 0.34–0.47 *p* < 0.001; I^2^ = 10%; Supplementary Figure S3].

Data on recurrent cerebral infarction were reported in seventeen trials (15–20, 29, 31, 34–41, 43). Recurrent cerebral infarction occurred in 156 participants (8.9%) in the DAPT group and 264 participants (19.1%) in the SAPT group. The findings indicated a significantly lower risk of recurrent cerebral infarction in individuals treated with DAPT [RR = 0.37; 95%CI: 0.30–0.44 *p* < 0.001; I^2^ = 2%; Supplementary Figure S4].

Thirteen trials included evaluations of NIHSS (15, 16, 28, 29, 31–37, 41, 42). These studies showed a notable reduction of NIHSS points in the short-term among patients treated with DAPT [MD = −3.33; 95%CI: −4.26–2.40 *p* < 0.001; I^2^ = 99%; Supplementary Figure S5].

Functional outcome as measured by mRS(3–6) was reported only in one trial (17). The findings indicated no significant difference in outcomes between the DAPT and SAPT groups [RR = 0.83; 95%CI: 0.66–1.03 *p* = 0.07; Supplementary Figure S6].

### Safety outcomes – bleeding events, cerebral hemorrhage, and death

Bleeding events were documented in nine trials (15–20, 29, 35, 43). In these studies, 91 cases (6.6%) of bleeding occurred in the DAPT group, compared to 50 cases (5.0%) in the SAPT group. The analysis demonstrated that DAPT did not significantly increase the risk of bleeding events [RR = 1.34; 95%CI: 0.97–1.85 *p* = 0.07; I^2^ = 37%; Supplementary Figure S7].

Cerebral hemorrhage outcomes were reported in five trials (17–20, 43). In these studies, cerebral hemorrhage was less common among participants treated with DAPT, affecting 6 participants (0.5%), compared to 8 participants (1.0%) in the SAPT group. Nonetheless, the meta-analysis revealed no statistically significant difference in the risk of cerebral hemorrhage between the two groups [RR = 0.47; 95%CI: 0.17–1.31 *p* = 0.15; I^2^ = 0%; Figure S8].

Death outcomes were detailed in five trials (16–18, 20, 43). Among these, 37 patients (3.0%) in the DAPT group and 30 patients (3.6%) in the SAPT group died. The analysis showed a significantly lower risk of death in the DAPT group than the SAPT group [RR = 0.62; 95%CI: 0.39–0.99 *p* = 0.05; I^2^ = 0%; Supplementary Figure S9].

### Subgroup analysis

#### Duration of DAPT

In subgroup analysis by DAPT duration, there was no significant difference in the incidence of composite endpoint events. However, DAPT greater than 2 months will reduce the occurrence of deaths (Supplementary Figures S10–S17).

#### Types of DAPT

The most common type of DAPT is the combination of aspirin and clopidogrel. We conducted a subgroup analysis by types of DAPTs. All types of DAPTs significantly reduced the incidences of composite endpoint events, recurrent stroke, ischemic events, and recurrent stroke. However, combination of aspirin and clopidogrel caused a higher risk of bleeding events and was not as effective as other DAPTs in reducing mortality (Supplementary Figures S18–S24).

## Discussion

Despite ongoing debates regarding the safety and efficacy of DAPT in ischemic stroke patients with ICAS, there is currently no consensus on its use (4, 5, 44). Our systematic review, incorporating recent RCTs, shows that DAPT significantly reduced the composite of ischemic and bleeding events, strokes, and cerebral infarctions in these patients. Furthermore, DAPT improved short-term neurological function and lowered mortality risk without increasing bleeding or cerebral hemorrhage events, highlighting its safety profile.

Antiplatelet drugs inhibit platelet adhesion, release, and aggregation by various means, including reducing the synthesis of thromboxane A2 by inhibiting cyclooxygenase-1 (COX-1), blocking the binding of adenosine diphosphate (ADP) to its platelet receptor to inhibit the activation of the glycoprotein IIb/IIIa complex mediated by ADP, inhibiting the activity of phosphodiesterase, and other mechanisms (30, 45, 46). DAPT inhibits platelet aggregation in two different mechanisms. The meta-analysis indicated that DAPT decreased the incidence of thrombosis and ischemic events more effectively than single antiplatelet therapy (SAPT). This aligns with the existing literature (15, 17, 18, 20, 43, 47), suggesting DAPT’s potential in reducing both short and long-term morbidity and mortality associated with ischemic stroke. However, the limited number of trials assessing long-term functional outcomes using scales like the modified Rankin Scale (mRS) underscores the need to conduct well-powered randomized controlled trials to evaluate these outcomes.

Our review addresses concerns regarding the potential increase in hemorrhagic risks associated with the use of DAPT in managing ischemic stroke patients with ICAS. Hemorrhage, particularly cerebral hemorrhage, is a significant concern with antiplatelet therapy. The primary mechanisms by which antiplatelet agents induce bleeding include reduced hemostatic function and local mucosal damage. Our findings indicate that DAPT does not significantly elevate the risk of hemorrhagic events or cerebral hemorrhage in patients with ICAS. This is consistent with findings from large-scale, high-quality randomized controlled trials (18, 47–50). The pooled analysis of two landmark trials, CHANCE (47) and POINT (51), suggested that DAPT reduced the risk of major ischemic events at 90 days without increasing the risk of major hemorrhages (52). Due to the low incidence of bleeding events and most of the studies included were of small sample size, the confidence interval of hemorrhagic events in the meta-analysis results was wide. It is suggested that large sample randomized controlled trials are still needed for further verification in the future.

The subgroup analysis of the optimal duration and combination for DAPT showed differences in meta-analysis results and heterogeneity in some outcomes. Future clinical research is expected to determine the most effective and safe duration and combination for DAPT.

In the present systematic review, all the included studies originated from Asia, with the majority conducted in China. Asians have a relatively high incidence of intracranial arterial stenosis, with an incidence of 9–65%, compared with 10–16% in Europe and the United States (5). There is a lack of data on other populations, which may be related to the low incidence in Europe and the United States. Further randomized controlled trials are needed to study the efficacy and safety of DAPT in other populations.

This systematic review is subject to limitations. Notably, variability in outcomes was observed, influenced by factors such as the ICAS location and severity, timing of DAPT initiation (44, 53, 54), and duration of therapy (4, 5, 44). These heterogeneities could impact our findings’ reliability, yet they not accounted for in our meta-analysis. Additionally, the majority of included studies were single-center and had limited power.

We know from previous large clinical trials that DAPTs are very effective for minor strokes with a NIHSS score of less than or equal to 5 (47, 51). We were unable to assess the impact on patients with minor strokes due to a lack of relevant data. This is one of the limitations of the systematic review.

**Figure 4:**
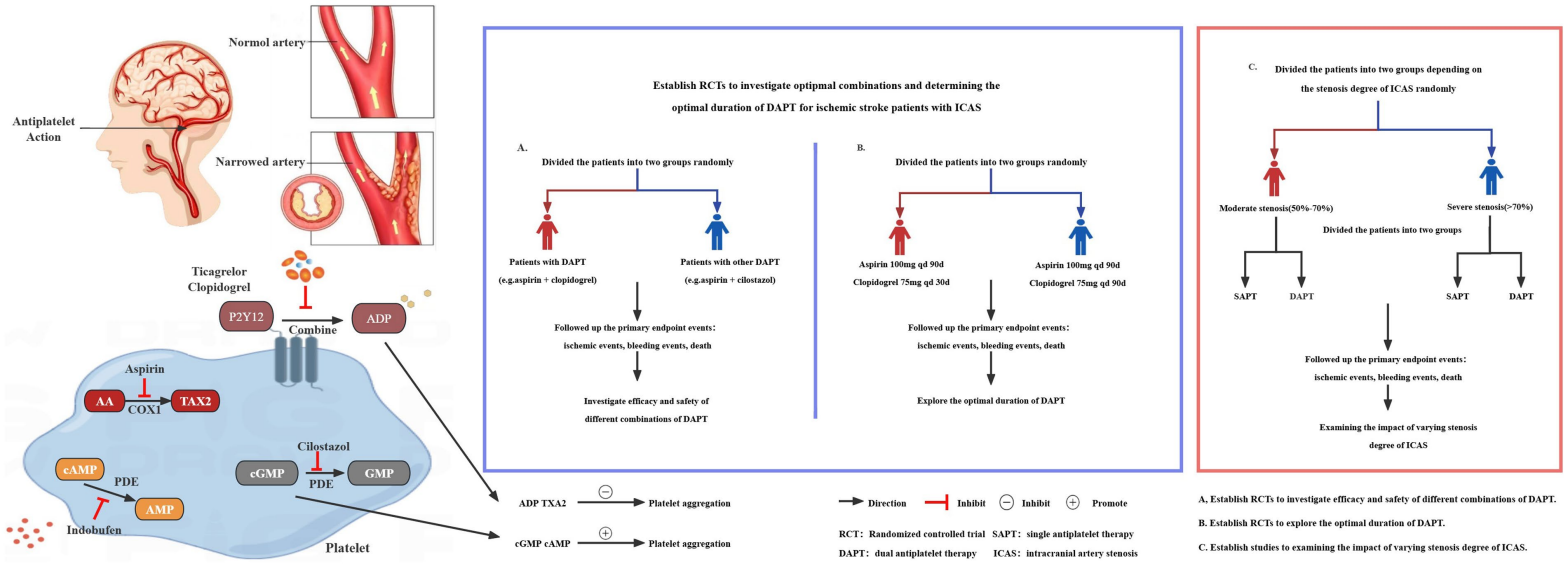
Key issues for future studies.

Our findings carry significant implications for clinical practice, public health, and future research. Given the limitations observed in the studies included in this review, future research should prioritize key areas: (1) identifying the most effective combinations and optimal duration for DAPT; (2) assessing how different degrees of ICAS stenosis affect outcomes; and (3) conducting multi-center, large-scale RCTs. Directions for future research are illustrated in Figure 4.

## Conclusion

Compared to SAPT, DAPT significantly lowered the risk of composite ischemic and bleeding events and stroke in patients with ICAS without raising the risk of bleeding or cerebral hemorrhage (54). Nonetheless, high-quality RCTs are urgently needed to confirm the efficacy and safety of DAPT in this patient population.

## Data availability statement

The original contributions presented in the study are included in the article/Supplementary material, further inquiries can be directed to the corresponding authors.

## Author contributions

HS: Writing – original draft. SH: Writing – original draft. PN: Writing – original draft. DZ: Writing – review & editing. NY: Writing – review & editing. QC: Writing – original draft. XL: Writing – review & editing. YL: Writing – original draft. SL: Writing – review & editing. JY: Writing – review & editing. XW: Writing – review & editing.
