# Supplementary material for: Dual antiplatelet therapy for ischemic stroke with intracranial arterial stenosis: a systematic review and meta-analysis
Source: Front Neurol. 2024 Jun 10;15:1411669. doi: 10.3389/fneur.2024.1411669 (PMC11194412; doi:10.3389/fneur.2024.1411669)
Supplement: Supplementary file 1 [file Image_1.pdf]

Supplemental figure to dual antiplatelet therapy for ischemic stroke with intracranial arterial stenosis: a systematic review and meta-analysis

Figure S1 Meta-analysis results for the effect of DAPT compared with SAPT on the composite endpoint of ischemic and bleeding events.
Figure S2 Meta-analysis results for the effect of DAPT compared with SAPT on recurrent stroke.
Figure S3 Meta-analysis results for the effect of DAPT compared with SAPT on ischemic events.
Figure S4 Meta-analysis results for the effect of DAPT compared with SAPT on recurrent cerebral infarction.
Figure S5 Meta-analysis results for the effect of DAPT compared with SAPT on NIHSS.
Figure S6 Meta-analysis results for the effect of DAPT compared with SAPT on mRS(3-6).
Figure S7 Meta-analysis results for the effect of DAPT compared with SAPT on bleeding events.
Figure S8 Meta-analysis results for the effect of DAPT compared with SAPT on cerebral hemorrhage.
Figure S9 Meta-analysis results for the effect of DAPT compared with SAPT on death.
Figure S10 Duration of DAPT subgroup analysis results for the effect of DAPT compared with SAPT on ischemic and bleeding events.
Figure S11 Duration of DAPT subgroup analysis results for the effect of DAPT compared with SAPT on recurrent stroke.
Figure S12 Duration of DAPT subgroup analysis results for the effect of DAPT compared with SAPT on ischemic events.
Figure S13 Duration of DAPT subgroup analysis results for the effect of DAPT compared with SAPT on recurrent cerebral infarction.
Figure S14 Duration of DAPT subgroup analysis results for the effect of DAPT compared with SAPT on bleeding events.
Figure S15 Duration of DAPT subgroup analysis results for the effect of DAPT compared with SAPT on cerebral hemorrhage.
Figure S16 Duration of DAPT subgroup analysis results for the effect of DAPT compared with SAPT on death.
Figure S17 Duration of DAPT subgroup analysis results for the effect of DAPT compared with SAPT on NIHSS.
Figure S18 Types of DAPT subgroup analysis results for the effect of DAPT compared with SAPT on ischemic and bleeding events.
Figure S19 Types of DAPT subgroup analysis results for the effect of DAPT

compared with SAPT on recurrent stroke.

Figure S20 Types of DAPT subgroup analysis results for the effect of DAPT compared with SAPT on ischemic events.

Figure S21 Types of DAPT subgroup analysis results for the effect of DAPT compared with SAPT on recurrent cerebral infarction.

Figure S22 Types of DAPT subgroup analysis results for the effect of DAPT compared with SAPT on bleeding events.

Figure S23 Types of DAPT subgroup analysis results for the effect of DAPT compared with SAPT on cerebral hemorrhage.

Figure S24 Types of DAPT subgroup analysis results for the effect of DAPT compared with SAPT on death.

Figures S1-S24

Study or Subgroup	DAPT Events	DAPT Total	SAPT Events	SAPT Total	Weight	Risk Ratio M-H, Fixed, 95% CI
Cheng 2016	1	43	8	43	1.8%	0.13 [0.02, 0.96]
Ge 2019	8	46	15	92	2.3%	1.07 [0.49, 2.33]
Hao 2021	9	40	18	80	2.8%	1.00 [0.49, 2.02]
Jiang 2011	1	49	7	40	1.8%	0.12 [0.01, 0.91]
Li, S 2015	0	31	5	31	1.3%	0.09 [0.01, 1.58]
Li, Y 2015	1	28	5	28	1.1%	0.20 [0.02, 1.60]
Liang 2015	2	57	9	57	2.1%	0.22 [0.05, 0.98]
Lin 2016	1	84	8	84	1.8%	0.13 [0.02, 0.98]
Liu, M 2017	9	34	22	34	5.0%	0.41 [0.22, 0.75]
Liu, Y 2021	2	30	6	30	1.4%	0.33 [0.07, 1.52]
Ma 2020	0	29	6	29	1.5%	0.08 [0.00, 1.31]
Qu 2013	6	26	14	27	3.1%	0.45 [0.20, 0.98]
Shinichiro 2015	9	83	12	80	2.8%	0.72 [0.32, 1.62]
Tai 2015	4	66	11	66	2.5%	0.36 [0.12, 1.08]
Wang, C 2015	34	284	47	286	10.7%	0.73 [0.48, 1.10]
Wang, L 2012	219	787	175	339	56.1%	0.54 [0.46, 0.63]
Wang, Y 2018	1	38	8	38	1.8%	0.13 [0.02, 0.95]
**Total (95% CI)**		**1755**		**1384**	**100.0%**	**0.52 [0.46, 0.59]**
Total events	307		376			

Heterogeneity: Chi² = 24.43, df = 16 (P = 0.08); I² = 34%
Test for overall effect: Z = 9.87 (P < 0.00001)

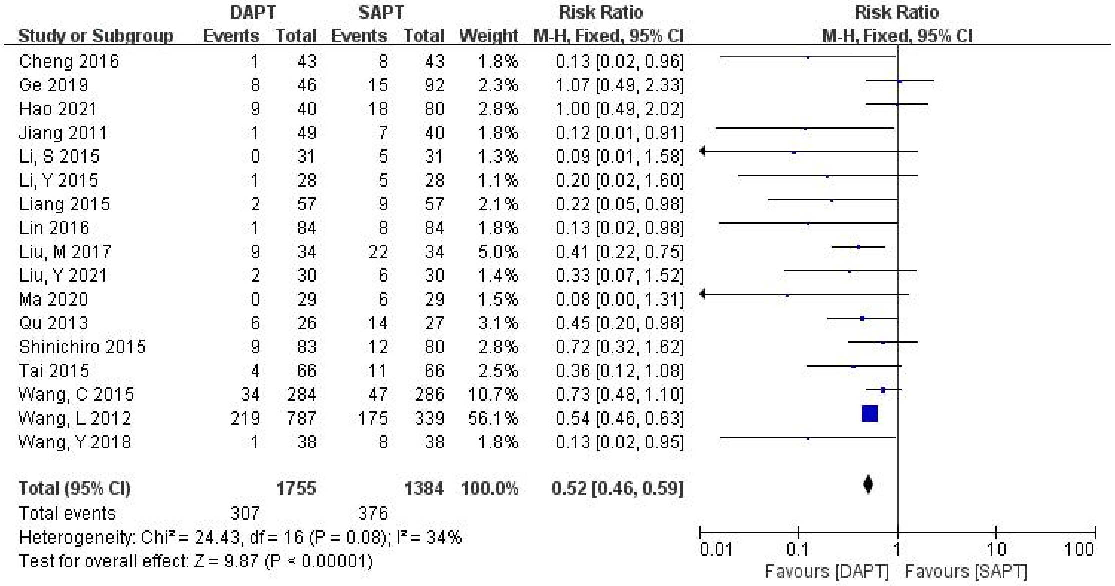

Supplementary Figure 1 Meta-analysis results for the effect of DAPT compared with SAPT on the composite endpoint of ischemic and bleeding events.

Study or Subgroup	DAPT Events	DAPT Total	SAPT Events	SAPT Total	Weight	Risk Ratio M-H, Fixed, 95% CI
Cheng 2016	1	43	8	43	2.6%	0.13 [0.02, 0.96]
Ge 2019	0	46	12	92	2.7%	0.08 [0.00, 1.31]
Hao 2021	0	40	13	80	2.9%	0.07 [0.00, 1.20]
Jiang 2011	1	49	4	40	1.4%	0.20 [0.02, 1.75]
Li, S 2015	0	31	5	31	1.8%	0.09 [0.01, 1.58]
Li, Y 2015	1	28	5	28	1.6%	0.20 [0.02, 1.60]
Liang 2015	2	57	9	57	2.9%	0.22 [0.05, 0.98]
Lin 2016	1	84	8	84	2.6%	0.13 [0.02, 0.98]
Liu, M 2017	8	34	22	34	7.1%	0.36 [0.19, 0.70]
Liu, Y 2021	1	30	6	30	1.9%	0.17 [0.02, 1.30]
Ma 2020	0	29	6	29	2.1%	0.08 [0.00, 1.31]
Qu 2013	6	26	14	27	4.4%	0.45 [0.20, 0.98]
Shinichiro 2015	4	83	8	80	2.6%	0.48 [0.15, 1.54]
Tai 2015	4	66	11	66	3.5%	0.36 [0.12, 1.08]
Wang, C 2015	6	284	19	286	6.1%	0.32 [0.13, 0.78]
Wang, L 2012	126	787	114	339	51.2%	0.48 [0.38, 0.59]
Wang, Y 2018	1	38	8	38	2.6%	0.13 [0.02, 0.95]
**Total (95% CI)**		**1755**		**1384**	**100.0%**	**0.37 [0.30, 0.44]**
Total events	162		272			

Heterogeneity: Chi² = 15.30, df = 16 (P = 0.50); I² = 0%
Test for overall effect: Z = 10.51 (P < 0.00001)

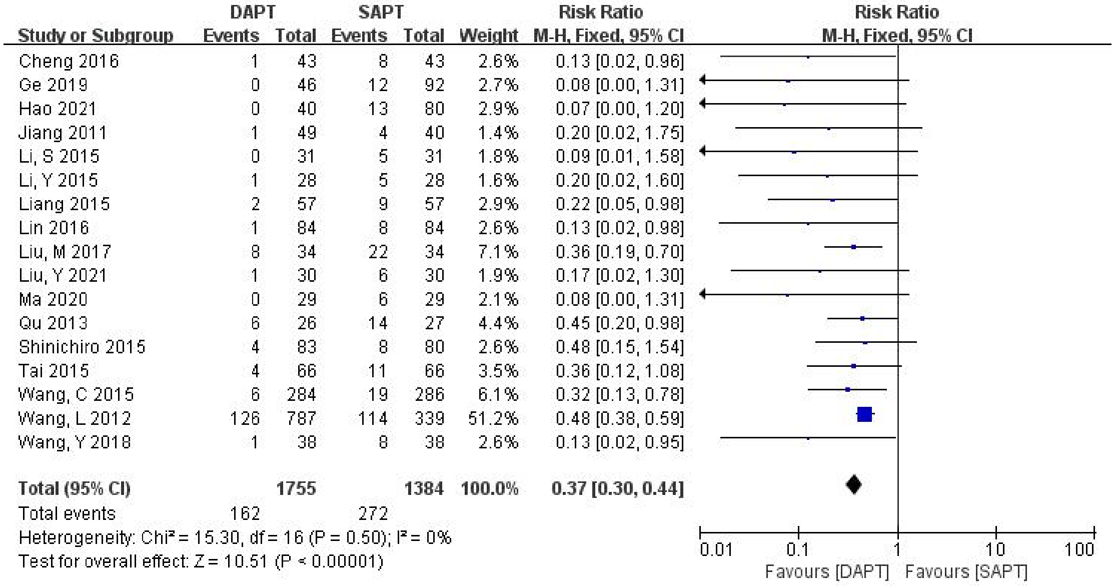

Supplementary Figure 2 Meta-analysis results for the effect of DAPT compared with SAPT on recurrent stroke.

Study or Subgroup	DAPT Events	DAPT Total	SAPT Events	SAPT Total	Weight	Risk Ratio M-H, Fixed, 95% CI
Cheng 2016	1	43	8	43	2.1%	0.13 [0.02, 0.96]
Ge 2019	0	46	12	92	2.2%	0.08 [0.00, 1.31]
Hao 2021	0	40	13	80	2.4%	0.07 [0.00, 1.20]
Jiang 2011	0	49	6	40	1.9%	0.06 [0.00, 1.09]
Li, S 2015	0	31	5	31	1.4%	0.09 [0.01, 1.58]
Li, Y 2015	1	28	5	28	1.3%	0.20 [0.02, 1.60]
Liang 2015	2	57	9	57	2.4%	0.22 [0.05, 0.98]
Lin 2016	1	84	8	84	2.1%	0.13 [0.02, 0.98]
Liu, M 2017	8	34	22	34	5.8%	0.36 [0.19, 0.70]
Liu, Y 2021	1	30	6	30	1.6%	0.17 [0.02, 1.30]
Ma 2020	0	29	6	29	1.7%	0.08 [0.00, 1.31]
Qu 2013	6	26	14	27	3.6%	0.45 [0.20, 0.98]
Shinichiro 2015	5	83	9	80	2.4%	0.54 [0.19, 1.53]
Tai 2015	4	66	11	66	2.9%	0.36 [0.12, 1.08]
Wang, C 2015	18	284	32	286	8.4%	0.57 [0.33, 0.99]
Wang, L 2012	168	787	152	339	55.8%	0.48 [0.40, 0.57]
Wang, Y 2018	1	38	8	38	2.1%	0.13 [0.02, 0.95]
**Total (95% CI)**		**1755**		**1384**	**100.0%**	**0.40 [0.34, 0.47]**
Total events	216		326			

Heterogeneity: Chi² = 17.84, df = 16 (P = 0.33); I² = 10%
Test for overall effect: Z = 11.39 (P < 0.00001)

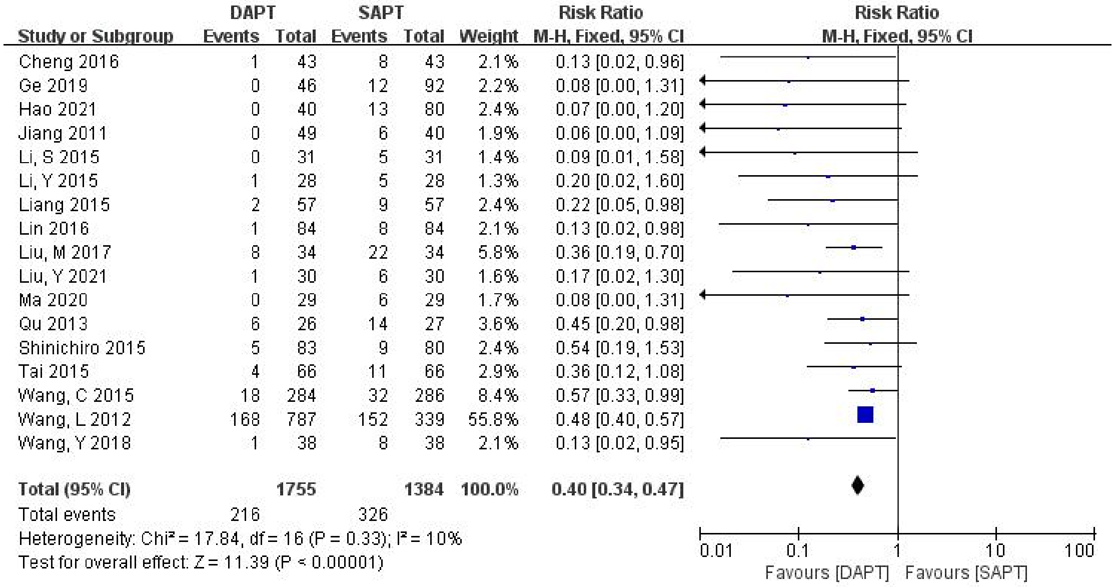

Supplemental Figure 3 Meta-analysis results for the effect of DAPT compared with SAPT on ischemic events.

Study or Subgroup	DAPT Events	DAPT Total	SAPT Events	SAPT Total	Weight	Risk Ratio M-H, Fixed, 95% CI
Cheng 2016	1	43	8	43	2.7%	0.13 [0.02, 0.96]
Ge 2019	0	46	12	92	2.8%	0.08 [0.00, 1.31]
Hao 2021	0	40	13	80	3.0%	0.07 [0.00, 1.20]
Jiang 2011	0	49	3	40	1.3%	0.12 [0.01, 2.20]
Li, S 2015	0	31	5	31	1.8%	0.09 [0.01, 1.58]
Li, Y 2015	1	28	5	28	1.7%	0.20 [0.02, 1.60]
Liang 2015	2	57	9	57	3.0%	0.22 [0.05, 0.98]
Lin 2016	1	84	8	84	2.7%	0.13 [0.02, 0.98]
Liu, M 2017	8	34	22	34	7.3%	0.36 [0.19, 0.70]
Liu, Y 2021	1	30	6	30	2.0%	0.17 [0.02, 1.30]
Ma 2020	0	29	6	29	2.2%	0.08 [0.00, 1.31]
Qu 2013	6	26	14	27	4.6%	0.45 [0.20, 0.98]
Shinichiro 2015	4	83	6	80	2.0%	0.64 [0.19, 2.19]
Tai 2015	4	66	11	66	3.6%	0.36 [0.12, 1.08]
Wang, C 2015	5	284	18	286	5.9%	0.28 [0.11, 0.74]
Wang, L 2012	122	787	110	339	50.9%	0.48 [0.38, 0.60]
Wang, Y 2018	1	38	8	38	2.7%	0.13 [0.02, 0.95]
**Total (95% CI)**		**1755**		**1384**	**100.0%**	**0.37 [0.30, 0.44]**
Total events	156		264			

Heterogeneity: Chi² = 16.40, df = 16 (P = 0.43); I² = 2%
Test for overall effect: Z = 10.35 (P < 0.00001)

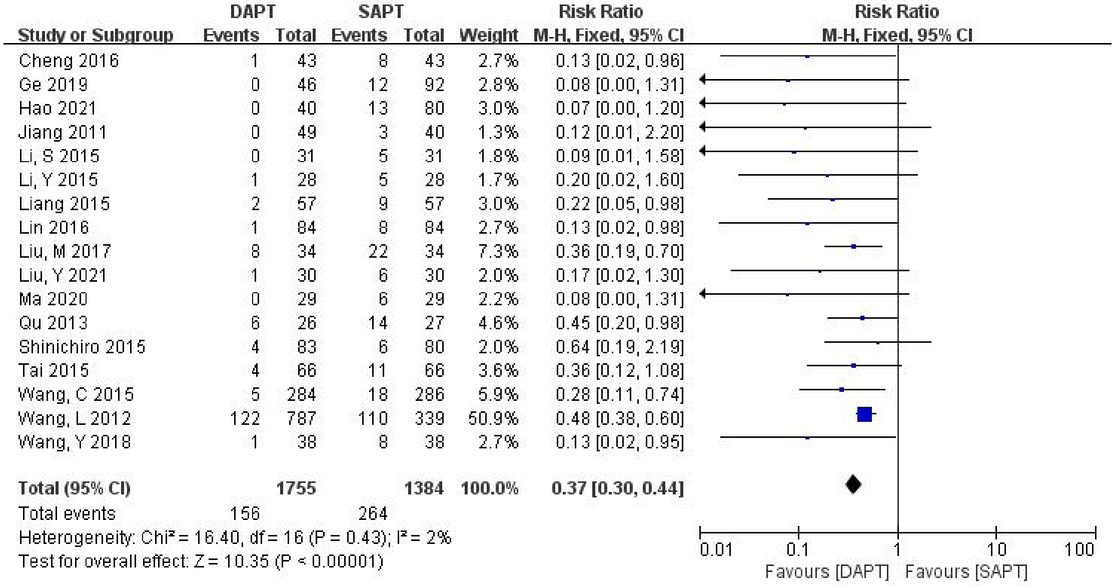

Supplemental Figure 4 Meta-analysis results for the effect of DAPT compared with SAPT on cerebral infarction occurrence.

Study or Subgroup	DAPT Mean	DAPT SD	DAPT Total	SAPT Mean	SAPT SD	SAPT Total	Weight	Mean Difference IV, Random, 95% CI
Cheng 2016	5.15	1.26	43	7.67	1.08	43	7.8%	-2.52 [-3.02, -2.02]
Fan 2020	3.11	0.34	59	5.48	1.21	59	7.9%	-2.37 [-2.69, -2.05]
Ge 2019	8.3	4.1	46	14.6	3.6	92	6.8%	-6.30 [-7.69, -4.91]
Gin 2020	6.21	1.63	42	14.59	1.57	42	7.7%	-8.38 [-9.06, -7.70]
Hao 2021	9.27	2.45	40	14.21	2.99	80	7.3%	-4.94 [-5.94, -3.94]
Li, S 2015	3	0.8	31	5.7	1.1	31	7.8%	-2.70 [-3.18, -2.22]
Lin 2016	3.2	0.5	84	5.3	0.7	84	8.0%	-2.10 [-2.28, -1.92]
Liu, M 2017	3.1	1.2	34	5.5	1.3	34	7.7%	-2.40 [-2.99, -1.81]
Liu, Y 2021	0.91	0.26	30	1.18	0.49	30	8.0%	-0.27 [-0.47, -0.07]
Ma 2020	6.15	1.08	29	8.24	1.02	29	7.8%	-2.09 [-2.63, -1.55]
Mao 2014	3.1	1.6	75	5.4	1.2	75	7.8%	-2.30 [-2.75, -1.85]
Wang, X 2021	8.76	1.42	50	13.94	1.57	50	7.7%	-5.18 [-5.77, -4.59]
Wang, Y 2018	3.27	1.19	38	5.65	1.34	38	7.8%	-2.38 [-2.95, -1.81]
**Total (95% CI)**			**601**			**687**	**100.0%**	**-3.33 [-4.26, -2.40]**

Heterogeneity: Tau² = 2.82; Chi² = 831.87, df = 12 (P < 0.00001); I² = 99%
Test for overall effect: Z = 7.00 (P < 0.00001)

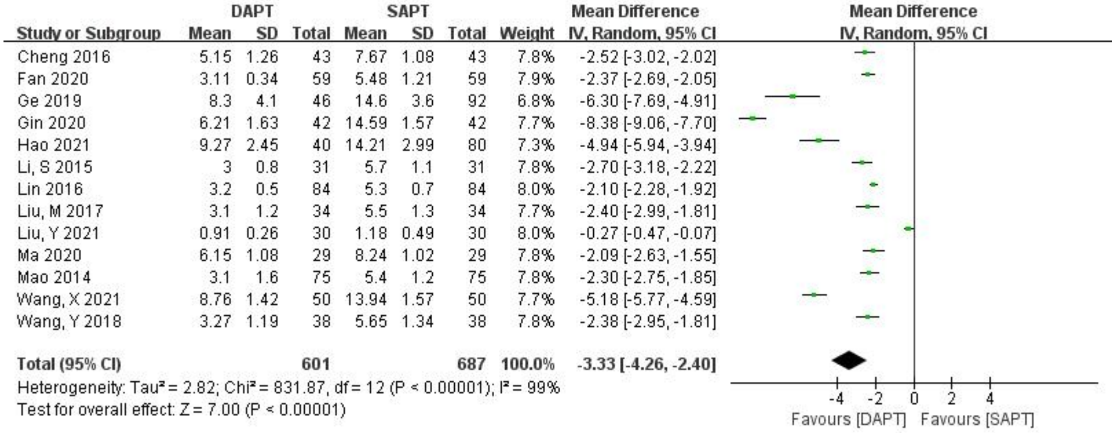

Supplemental Figure 5 Meta-analysis results for the effect of DAPT compared with SAPT on NIHSS.

Study or Subgroup	DAPT Events	DAPT Total	SAPT Events	SAPT Total	Weight	Risk Ratio M-H, Fixed, 95% CI
Wang, C 2015	91	284	111	286	100.0%	0.83 [0.66, 1.03]
**Total (95% CI)**		**284**		**286**	**100.0%**	**0.83 [0.66, 1.03]**
Total events	91		111			

Heterogeneity: Not applicable
Test for overall effect: Z = 1.68 (P = 0.09)

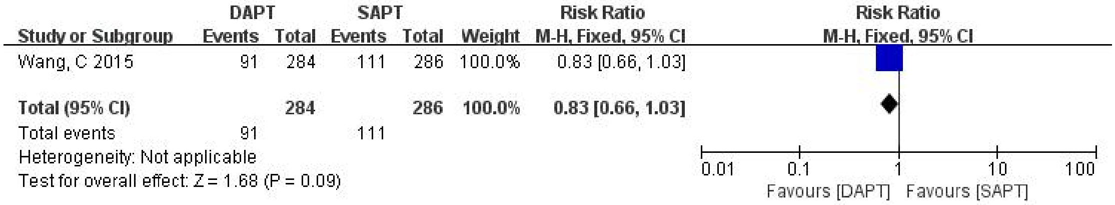

Supplemental Figure 6 Meta-analysis results for the effect of DAPT compared with SAPT on mRS(3-6).

Study or Subgroup	DAPT Events	DAPT Total	SAPT Events	SAPT Total	Weight	Risk Ratio M-H, Fixed, 95% CI
Ge 2019	8	46	3	92	3.5%	5.33 [1.48, 19.16]
Hao 2021	9	40	5	80	5.8%	3.60 [1.29, 10.04]
Jiang 2011	1	49	1	40	1.9%	0.82 [0.05, 12.64]
Liu, M 2017	1	34	0	34	0.9%	3.00 [0.13, 71.15]
Liu, Y 2021	1	30	0	30	0.9%	3.00 [0.13, 70.83]
Qu 2013	0	26	0	27		Not estimable
Shinichiro 2015	4	83	3	80	5.3%	1.29 [0.30, 5.56]
Wang, C 2015	16	284	15	286	26.0%	1.07 [0.54, 2.13]
Wang, L 2012	51	787	23	339	55.8%	0.96 [0.59, 1.54]
**Total (95% CI)**		**1379**		**1008**	**100.0%**	**1.34 [0.97, 1.85]**
Total events	91		50			

Heterogeneity: Chi² = 11.03, df = 7 (P = 0.14); I² = 37%
Test for overall effect: Z = 1.79 (P = 0.07)

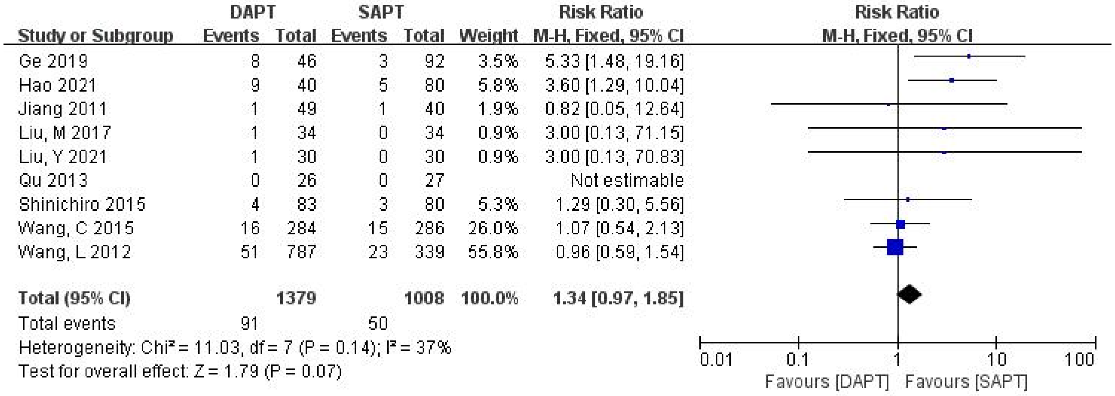

Supplementary Figure 7 Meta-analysis results for the effect of DAPT compared with SAPT on bleeding events.

Study or Subgroup	DAPT Events	DAPT Total	SAPT Events	SAPT Total	Weight	Risk Ratio M-H, Fixed, 95% CI
Jiang 2011	1	49	1	40	10.8%	0.82 [0.05, 12.64]
Qu 2013	0	26	0	27		Not estimable
Shinichiro 2015	0	83	2	80	24.9%	0.19 [0.01, 3.96]
Wang, C 2015	1	284	1	286	9.7%	1.01 [0.06, 16.02]
Wang, L 2012	4	787	4	339	54.6%	0.43 [0.11, 1.71]
**Total (95% CI)**		**1229**		**772**	**100.0%**	**0.47 [0.17, 1.31]**
Total events	6		8			

Heterogeneity: Chi² = 0.80, df = 3 (P = 0.85); I² = 0%
Test for overall effect: Z = 1.44 (P = 0.15)

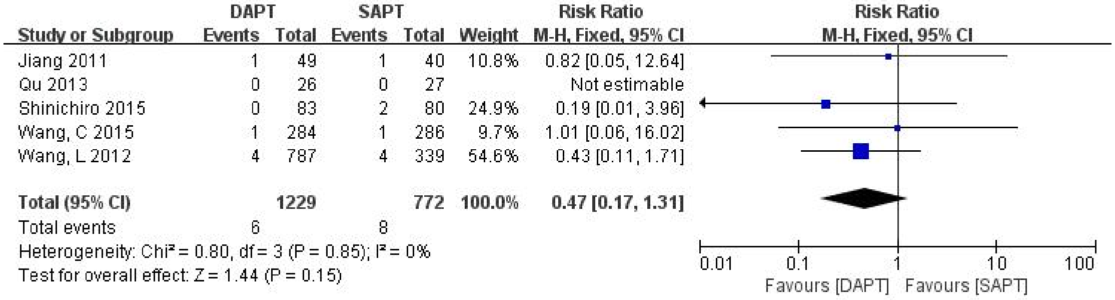

Supplementary Figure 8 Meta-analysis results for the effect of DAPT compared with SAPT on cerebral hemorrhage.

Study or Subgroup	DAPT Events	DAPT Total	SAPT Events	SAPT Total	Weight	Risk Ratio M-H, Fixed, 95% CI
Ge 2019	1	46	3	92	5.1%	0.67 [0.07, 6.23]
Jiang 2011	1	49	0	40	1.4%	2.46 [0.10, 58.79]
Shinichiro 2015	0	83	0	80		Not estimable
Wang, C 2015	2	284	2	286	5.0%	1.01 [0.14, 7.10]
Wang, L 2012	33	787	25	339	88.5%	0.57 [0.34, 0.94]
**Total (95% CI)**		**1249**		**837**	**100.0%**	**0.62 [0.39, 0.99]**
Total events	37		30			

Heterogeneity: Chi² = 1.08, df = 3 (P = 0.78); I² = 0%
Test for overall effect: Z = 1.99 (P = 0.05)

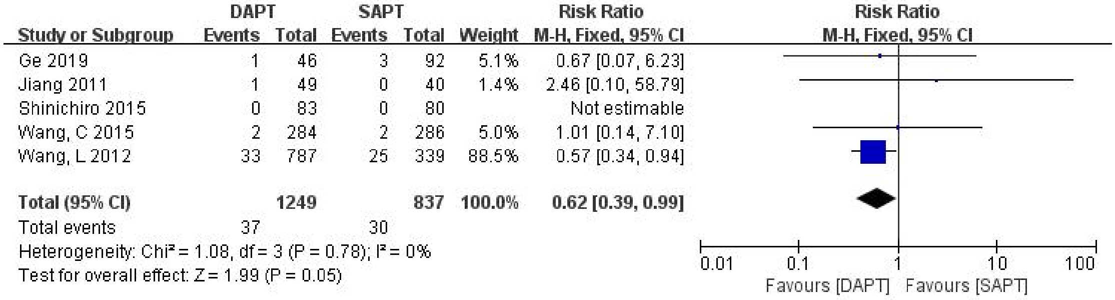

Supplemental Figure 9 Meta-analysis results for the effect of DAPT compared with SAPT on death.

Study or Subgroup	DAPT Events	DAPT Total	SAPT Events	SAPT Total	Weight	Risk Ratio M-H, Fixed, 95% CI
**1.1.1 ≤ 2 months**						
Hao 2021	9	40	18	80	2.8%	1.00 [0.49, 2.02]
Li, S 2015	0	31	5	31	1.3%	0.09 [0.01, 1.58]
Li, Y 2015	1	28	5	28	1.2%	0.20 [0.02, 1.60]
Liang 2015	2	57	9	57	2.1%	0.22 [0.05, 0.98]
Lin 2016	1	84	8	84	1.9%	0.13 [0.02, 0.98]
Liu, M 2017	8	34	22	34	5.1%	0.36 [0.19, 0.70]
Tai 2015	4	66	11	66	2.6%	0.36 [0.12, 1.08]
Wang, C 2015	34	284	47	286	10.9%	0.73 [0.48, 1.10]
Wang, Y 2018	1	38	8	38	1.9%	0.13 [0.02, 0.95]
**Subtotal (95% CI)**		**662**		**704**	**29.7%**	**0.50 [0.38, 0.66]**
Total events	60		133			
Heterogeneity: Chi² = 15.00, df = 8 (P = 0.06); I² = 47%						
Test for overall effect: Z = 4.92 (P < 0.00001)						
**1.1.2 > 2 months**						
Ge 2019	8	46	15	92	2.3%	1.07 [0.49, 2.33]
Jiang 2011	1	49	7	40	1.8%	0.12 [0.01, 0.91]
Liu, Y 2021	2	30	6	30	1.4%	0.33 [0.07, 1.52]
Ma 2020	0	29	6	29	1.5%	0.08 [0.00, 1.31]
Qu 2013	6	26	14	27	3.2%	0.45 [0.20, 0.98]
Shinichiro 2015	9	83	12	80	2.9%	0.72 [0.32, 1.62]
Wang, L 2012	219	787	175	339	57.1%	0.54 [0.46, 0.63]
**Subtotal (95% CI)**		**1050**		**637**	**70.3%**	**0.53 [0.46, 0.62]**
Total events	245		235			
Heterogeneity: Chi² = 8.04, df = 6 (P = 0.24); I² = 25%						
Test for overall effect: Z = 8.44 (P < 0.00001)						
**Total (95% CI)**		**1712**		**1341**	**100.0%**	**0.52 [0.46, 0.60]**
Total events	305		368			

Heterogeneity: Chi² = 22.90, df = 15 (P = 0.09); I² = 35%
Test for overall effect: Z = 9.69 (P < 0.00001)
Test for subgroup differences: Chi² = 0.18, df = 1 (P = 0.67), I² = 0%

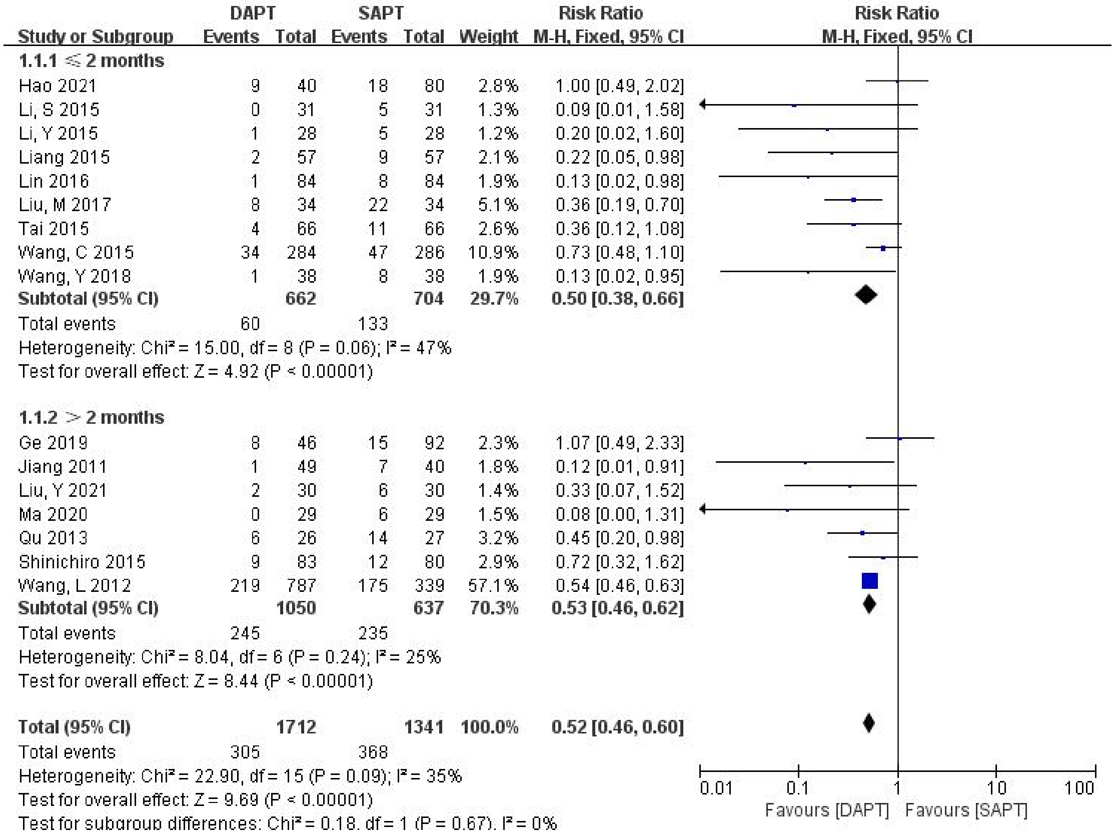

Supplemental Figure 10 Duration of DAPT subgroup analysis results for the effect of DAPT compared with SAPT on ischemic and bleeding events.

Study or Subgroup	DAPT Events	DAPT Total	SAPT Events	SAPT Total	Weight	Risk Ratio M-H, Random, 95% CI
**1.2.1 ≤ 2 months**						
Hao 2021	0	40	13	80	0.4%	0.07 [0.00, 1.20]
Li, S 2015	0	31	5	31	0.4%	0.09 [0.01, 1.58]
Li, Y 2015	1	28	5	28	0.8%	0.20 [0.02, 1.60]
Liang 2015	2	57	9	57	1.5%	0.22 [0.05, 0.98]
Lin 2016	1	84	8	84	0.8%	0.13 [0.02, 0.98]
Liu, M 2017	8	34	22	34	7.9%	0.36 [0.19, 0.70]
Tai 2015	4	66	11	66	2.8%	0.36 [0.12, 1.08]
Wang, C 2015	6	284	19	286	4.1%	0.32 [0.13, 0.78]
Wang, Y 2018	1	38	8	38	0.8%	0.13 [0.02, 0.95]
**Subtotal (95% CI)**		**662**		**704**	**19.6%**	**0.29 [0.19, 0.43]**
Total events	23		100			
Heterogeneity: Tau² = 0.00; Chi² = 4.14, df = 8 (P = 0.84); I² = 0%						
Test for overall effect: Z = 5.93 (P < 0.00001)						
**1.2.2 > 2 months**						
Ge 2019	0	46	12	92	0.4%	0.08 [0.00, 1.31]
Jiang 2011	1	49	4	40	0.7%	0.20 [0.02, 1.75]
Liu, Y 2021	1	30	6	30	0.8%	0.17 [0.02, 1.30]
Ma 2020	0	29	6	29	0.4%	0.08 [0.00, 1.31]
Qu 2013	6	26	14	27	5.4%	0.45 [0.20, 0.98]
Shinichiro 2015	4	83	8	80	2.5%	0.48 [0.15, 1.54]
Wang, L 2012	126	787	114	339	70.2%	0.48 [0.38, 0.59]
**Subtotal (95% CI)**		**1050**		**637**	**80.4%**	**0.46 [0.37, 0.56]**
Total events	138		164			
Heterogeneity: Tau² = 0.00; Chi² = 4.95, df = 6 (P = 0.55); I² = 0%						
Test for overall effect: Z = 7.51 (P < 0.00001)						
**Total (95% CI)**		**1712**		**1341**	**100.0%**	**0.42 [0.35, 0.50]**
Total events	161		264			
Heterogeneity: Tau² = 0.00; Chi² = 13.76, df = 15 (P = 0.54); I² = 0%						
Test for overall effect: Z = 9.35 (P < 0.00001)						
Test for subgroup differences: Chi² = 3.99, df = 1 (P = 0.05), I² = 74.9%						

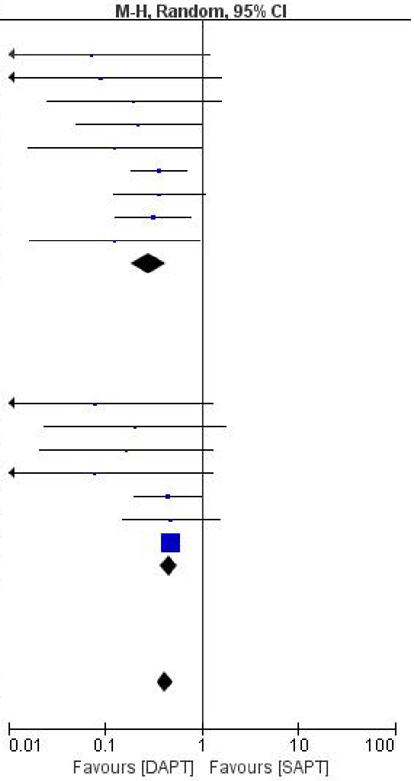

Supplemental Figure 11 Duration of DAPT subgroup analysis results for the effect of DAPT compared with SAPT on recurrent stroke.

Study or Subgroup	DAPT Events	DAPT Total	SAPT Events	SAPT Total	Weight	Risk Ratio M-H, Fixed, 95% CI
**1.3.1 ≤ 2 months**						
Hao 2021	0	40	13	80	2.4%	0.07 [0.00, 1.20]
Li, S 2015	0	31	5	31	1.5%	0.09 [0.01, 1.58]
Li, Y 2015	1	28	5	28	1.3%	0.20 [0.02, 1.60]
Liang 2015	2	57	9	57	2.4%	0.22 [0.05, 0.98]
Lin 2016	1	84	8	84	2.1%	0.13 [0.02, 0.98]
Liu, M 2017	8	34	22	34	5.9%	0.36 [0.19, 0.70]
Tai 2015	4	66	11	66	3.0%	0.36 [0.12, 1.08]
Wang, C 2015	18	284	32	286	8.6%	0.57 [0.33, 0.99]
Wang, Y 2018	1	38	8	38	2.1%	0.13 [0.02, 0.95]
**Subtotal (95% CI)**		**662**		**704**	**29.4%**	**0.33 [0.23, 0.47]**
Total events	35		113			
Heterogeneity: Chi² = 7.88, df = 8 (P = 0.45); I² = 0%						
Test for overall effect: Z = 6.09 (P < 0.00001)						
**1.3.2 > 2 months**						
Ge 2019	0	46	12	92	2.3%	0.08 [0.00, 1.31]
Jiang 2011	0	49	6	40	1.9%	0.06 [0.00, 1.09]
Liu, Y 2021	1	30	6	30	1.6%	0.17 [0.02, 1.30]
Ma 2020	0	29	6	29	1.7%	0.08 [0.00, 1.31]
Qu 2013	6	26	14	27	3.7%	0.45 [0.20, 0.98]
Shinichiro 2015	5	83	9	80	2.5%	0.54 [0.19, 1.53]
Wang, L 2012	168	787	152	339	57.0%	0.48 [0.40, 0.57]
**Subtotal (95% CI)**		**1050**		**637**	**70.6%**	**0.44 [0.37, 0.52]**
Total events	180		205			
Heterogeneity: Chi² = 6.56, df = 6 (P = 0.36); I² = 9%						
Test for overall effect: Z = 9.39 (P < 0.00001)						
**Total (95% CI)**		**1712**		**1341**	**100.0%**	**0.41 [0.35, 0.47]**
Total events	215		318			
Heterogeneity: Chi² = 16.10, df = 15 (P = 0.38); I² = 7%						
Test for overall effect: Z = 11.19 (P < 0.00001)						
Test for subgroup differences: Chi² = 1.86, df = 1 (P = 0.17), I² = 46.1%						

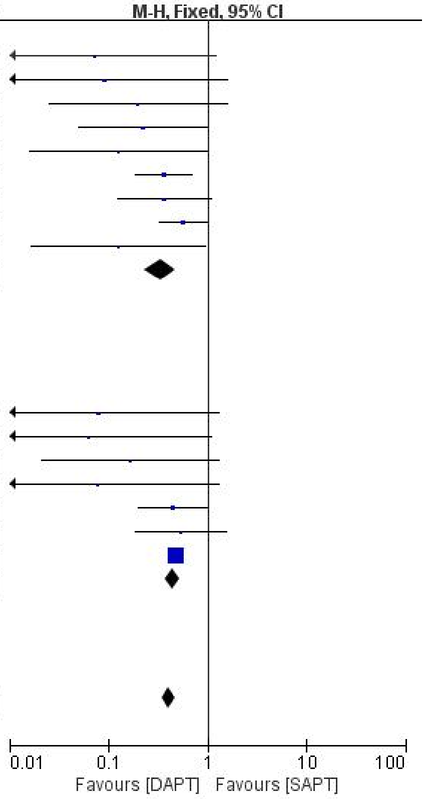

Supplemental Figure 12 Duration of DAPT subgroup analysis results for the effect of DAPT compared with SAPT on ischemic events.

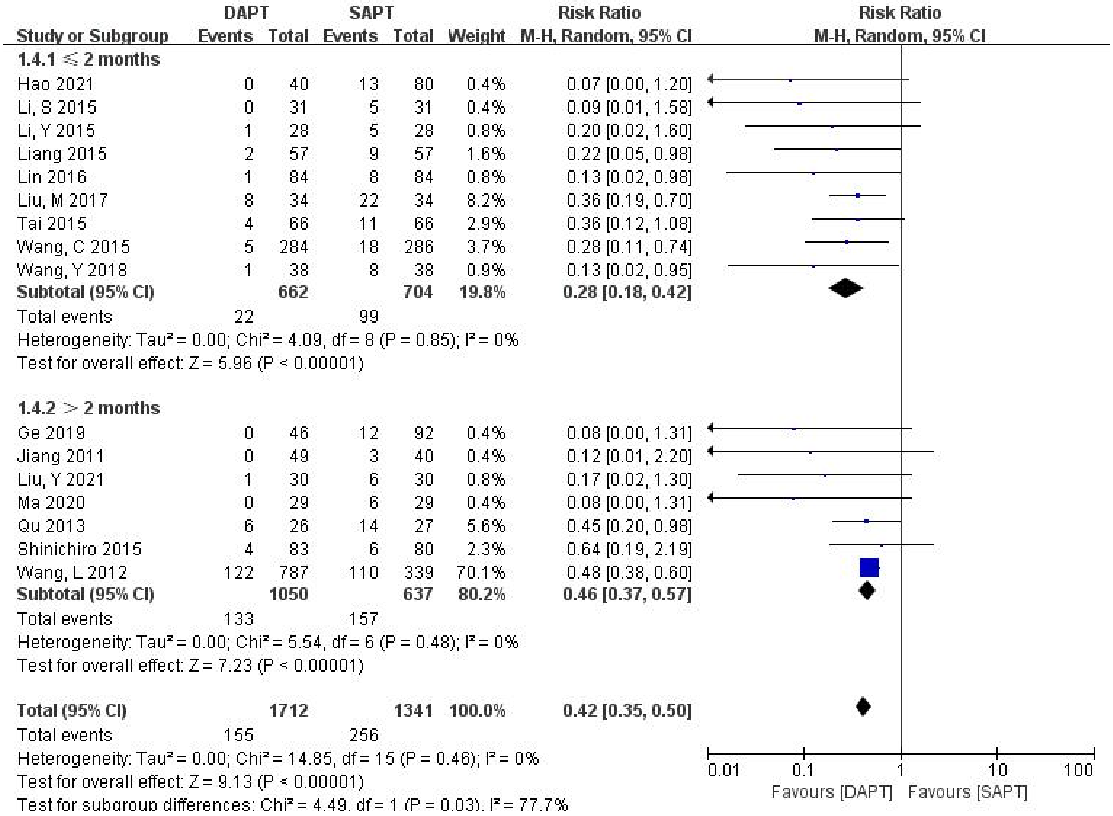

Supplemental Figure 13 Duration of DAPT subgroup analysis results for the effect of DAPT compared with SAPT on recurrent cerebral infarction.

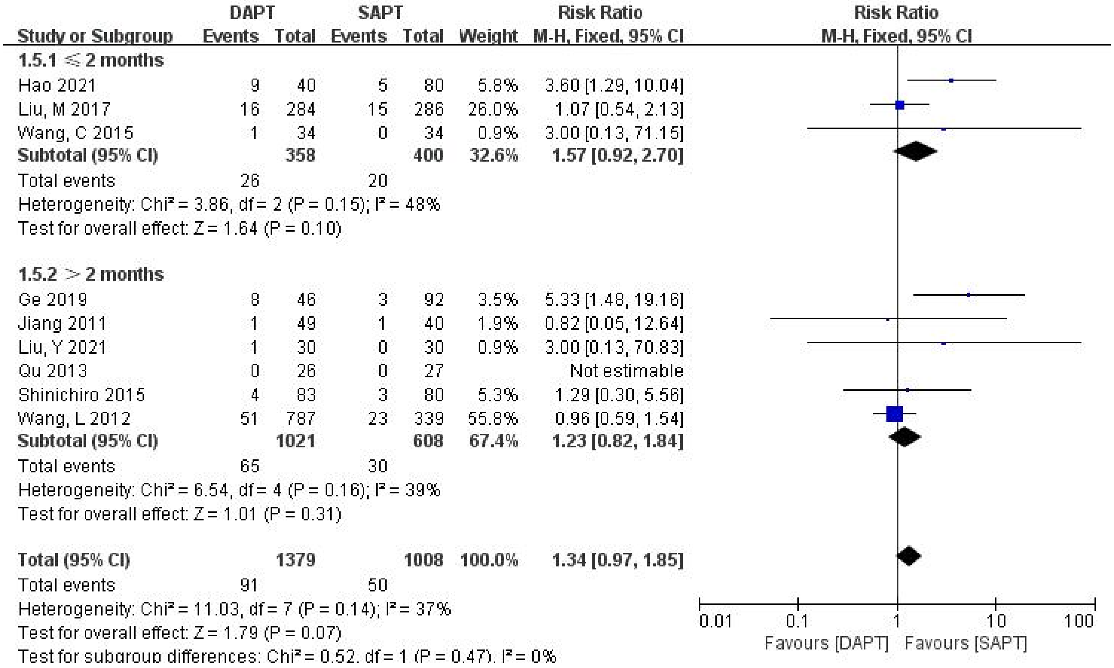

Supplemental Figure 14 Duration of DAPT subgroup analysis results for the effect

of DAPT compared with SAPT on bleeding events.

Study or Subgroup	DAPT Events	DAPT Total	SAPT Events	SAPT Total	Weight	Risk Ratio M-H, Fixed, 95% CI
**1.6.1 ≤ 2 months**						
Wang, C 2015	1	284	1	286	9.7%	1.01 [0.06, 16.02]
**Subtotal (95% CI)**		**284**		**286**	**9.7%**	**1.01 [0.06, 16.02]**
Total events	1		1			
Heterogeneity: Not applicable						
Test for overall effect: Z = 0.00 (P = 1.00)						
**1.6.2 > 2 months**						
Jiang 2011	1	49	1	40	10.8%	0.82 [0.05, 12.64]
Qu 2013	0	26	0	27		Not estimable
Shinichiro 2015	0	83	2	80	24.9%	0.19 [0.01, 3.96]
Wang, L 2012	4	787	4	339	54.6%	0.43 [0.11, 1.71]
**Subtotal (95% CI)**		**945**		**486**	**90.3%**	**0.41 [0.13, 1.27]**
Total events	5		7			
Heterogeneity: Chi² = 0.49, df = 2 (P = 0.78); I² = 0%						
Test for overall effect: Z = 1.55 (P = 0.12)						
**Total (95% CI)**		**1229**		**772**	**100.0%**	**0.47 [0.17, 1.31]**
Total events	6		8			
Heterogeneity: Chi² = 0.80, df = 3 (P = 0.85); I² = 0%						
Test for overall effect: Z = 1.44 (P = 0.15)						
Test for subgroup differences: Chi² = 0.35, df = 1 (P = 0.56), I² = 0%						

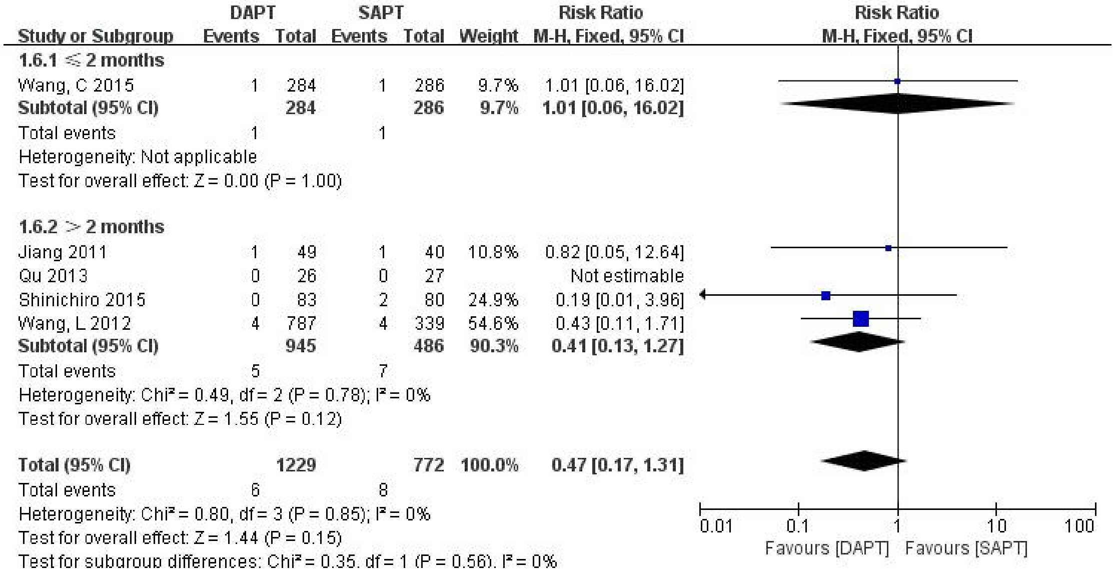

Supplemental Figure 15 Duration of DAPT subgroup analysis results for the effect of DAPT compared with SAPT on cerebral hemorrhage.

Study or Subgroup	DAPT Events	DAPT Total	SAPT Events	SAPT Total	Weight	Risk Ratio M-H, Fixed, 95% CI
**1.7.1 ≤ 2 months**						
Wang, C 2015	2	284	2	286	5.0%	1.01 [0.14, 7.10]
**Subtotal (95% CI)**		**284**		**286**	**5.0%**	**1.01 [0.14, 7.10]**
Total events	2		2			
Heterogeneity: Not applicable						
Test for overall effect: Z = 0.01 (P = 0.99)						
**1.7.2 > 2 months**						
Ge 2019	1	46	3	92	5.1%	0.67 [0.07, 6.23]
Jiang 2011	1	49	0	40	1.4%	2.46 [0.10, 58.79]
Shinichiro 2015	0	83	0	80		Not estimable
Wang, L 2012	33	787	25	339	88.5%	0.57 [0.34, 0.94]
**Subtotal (95% CI)**		**965**		**551**	**95.0%**	**0.60 [0.37, 0.97]**
Total events	35		28			
Heterogeneity: Chi² = 0.81, df = 2 (P = 0.67); I² = 0%						
Test for overall effect: Z = 2.06 (P = 0.04)						
**Total (95% CI)**		**1249**		**837**	**100.0%**	**0.62 [0.39, 0.99]**
Total events	37		30			
Heterogeneity: Chi² = 1.08, df = 3 (P = 0.78); I² = 0%						
Test for overall effect: Z = 1.99 (P = 0.05)						
Test for subgroup differences: Chi² = 0.25, df = 1 (P = 0.62), I² = 0%						

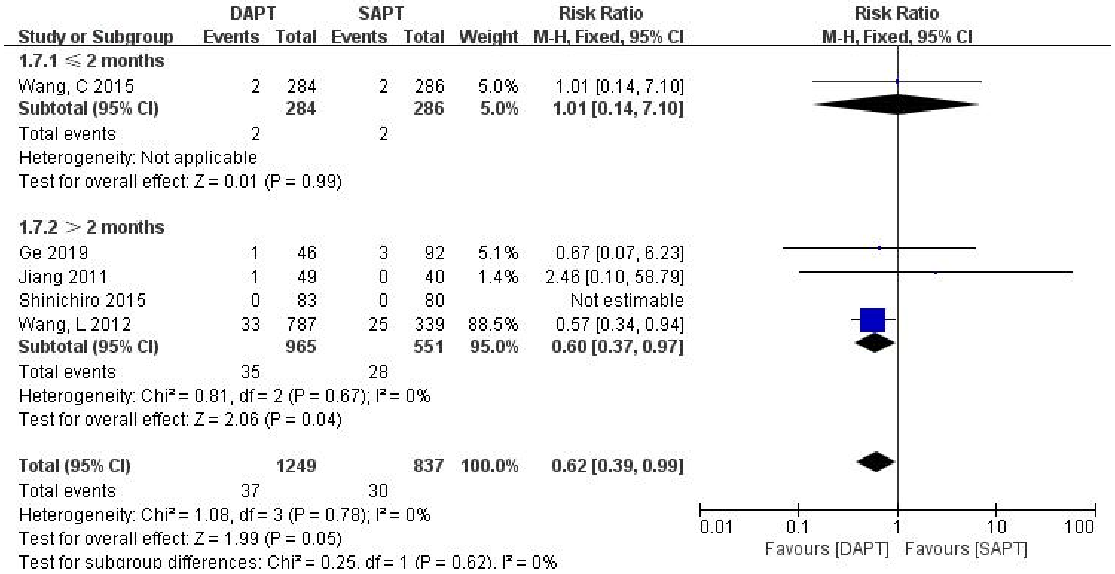

Supplemental Figure 16 Duration of DAPT subgroup analysis results for the effect of DAPT compared with SAPT on death.

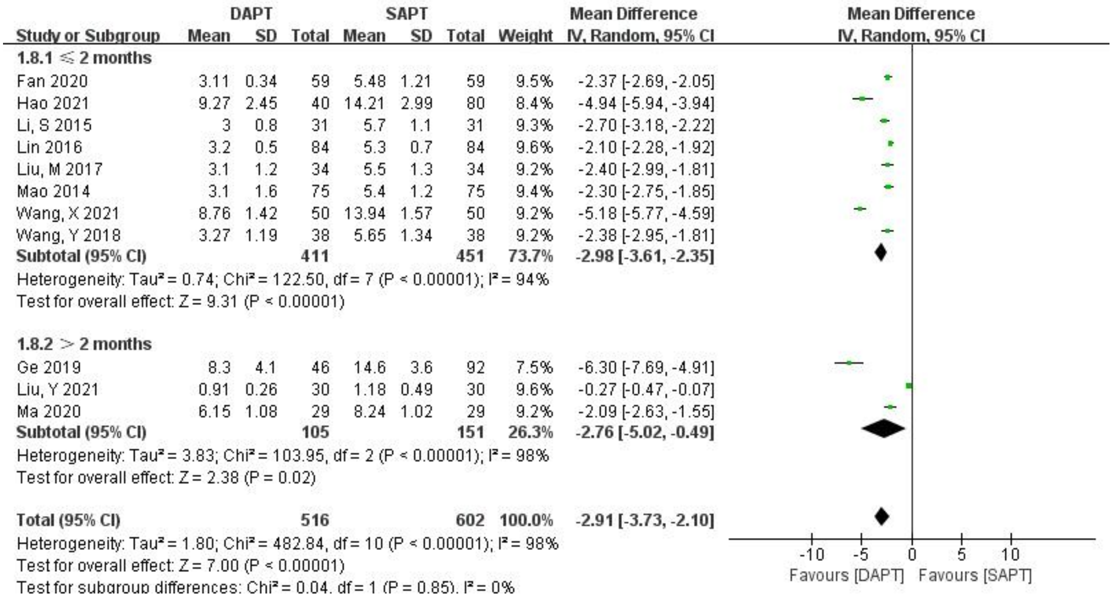

Supplemental Figure 17 Duration of DAPT subgroup analysis results for the effect of DAPT compared with SAPT on NIHSS.

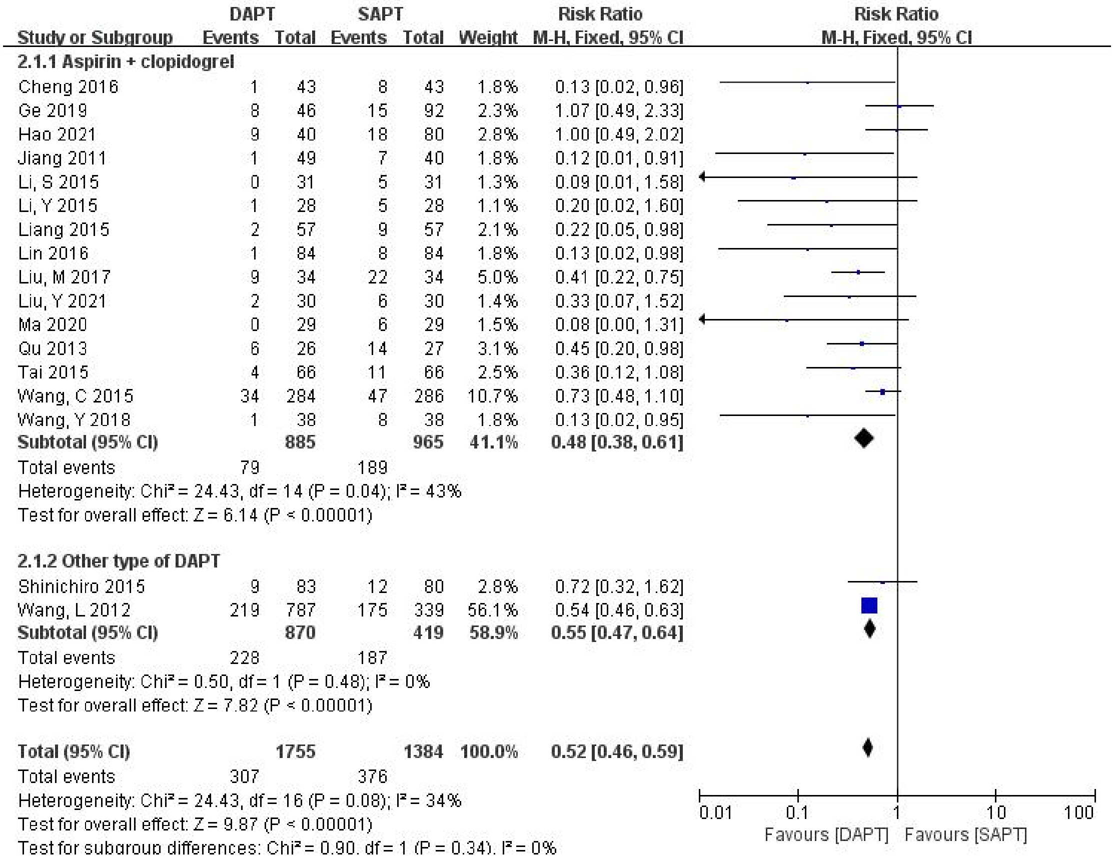

Supplemental Figure 18 Types of DAPT subgroup analysis results for the effect of DAPT compared with SAPT on ischemic and bleeding events.

Study or Subgroup	DAPT Events	DAPT Total	SAPT Events	SAPT Total	Weight	Risk Ratio M-H, Random, 95% CI
**2.2.1 Aspirin + clopidogrel**						
Cheng 2016	1	43	8	43	0.8%	0.13 [0.02, 0.96]
Ge 2019	0	46	12	92	0.4%	0.08 [0.00, 1.31]
Hao 2021	0	40	13	80	0.4%	0.07 [0.00, 1.20]
Jiang 2011	1	49	4	40	0.7%	0.20 [0.02, 1.75]
Li, S 2015	0	31	5	31	0.4%	0.09 [0.01, 1.58]
Li, Y 2015	1	28	5	28	0.8%	0.20 [0.02, 1.60]
Liang 2015	2	57	9	57	1.5%	0.22 [0.05, 0.98]
Lin 2016	1	84	8	84	0.8%	0.13 [0.02, 0.98]
Liu, M 2017	8	34	22	34	7.8%	0.36 [0.19, 0.70]
Liu, Y 2021	1	30	6	30	0.8%	0.17 [0.02, 1.30]
Ma 2020	0	29	6	29	0.4%	0.08 [0.00, 1.31]
Qu 2013	6	26	14	27	5.3%	0.45 [0.20, 0.98]
Tai 2015	4	66	11	66	2.8%	0.36 [0.12, 1.08]
Wang, C 2015	6	284	19	286	4.1%	0.32 [0.13, 0.78]
Wang, Y 2018	1	38	8	38	0.8%	0.13 [0.02, 0.95]
**Subtotal (95% CI)**		**885**		**965**	**27.9%**	**0.28 [0.20, 0.40]**
Total events	32		150			
Heterogeneity: Tau² = 0.00; Chi² = 8.57, df = 14 (P = 0.86); I² = 0%						
Test for overall effect: Z = 7.12 (P < 0.00001)						
**2.2.2 Other type of DAPT**						
Shinichiro 2015	4	83	8	80	2.5%	0.48 [0.15, 1.54]
Wang, L 2012	126	787	114	339	69.6%	0.48 [0.38, 0.59]
**Subtotal (95% CI)**		**870**		**419**	**72.1%**	**0.48 [0.38, 0.59]**
Total events	130		122			
Heterogeneity: Tau² = 0.00; Chi² = 0.00, df = 1 (P = 0.98); I² = 0%						
Test for overall effect: Z = 6.75 (P < 0.00001)						
**Total (95% CI)**		**1755**		**1384**	**100.0%**	**0.41 [0.34, 0.50]**
Total events	162		272			
Heterogeneity: Tau² = 0.00; Chi² = 15.30, df = 16 (P = 0.50); I² = 0%						
Test for overall effect: Z = 9.49 (P < 0.00001)						
Test for subgroup differences: Chi² = 6.12, df = 1 (P = 0.01), I² = 83.7%						

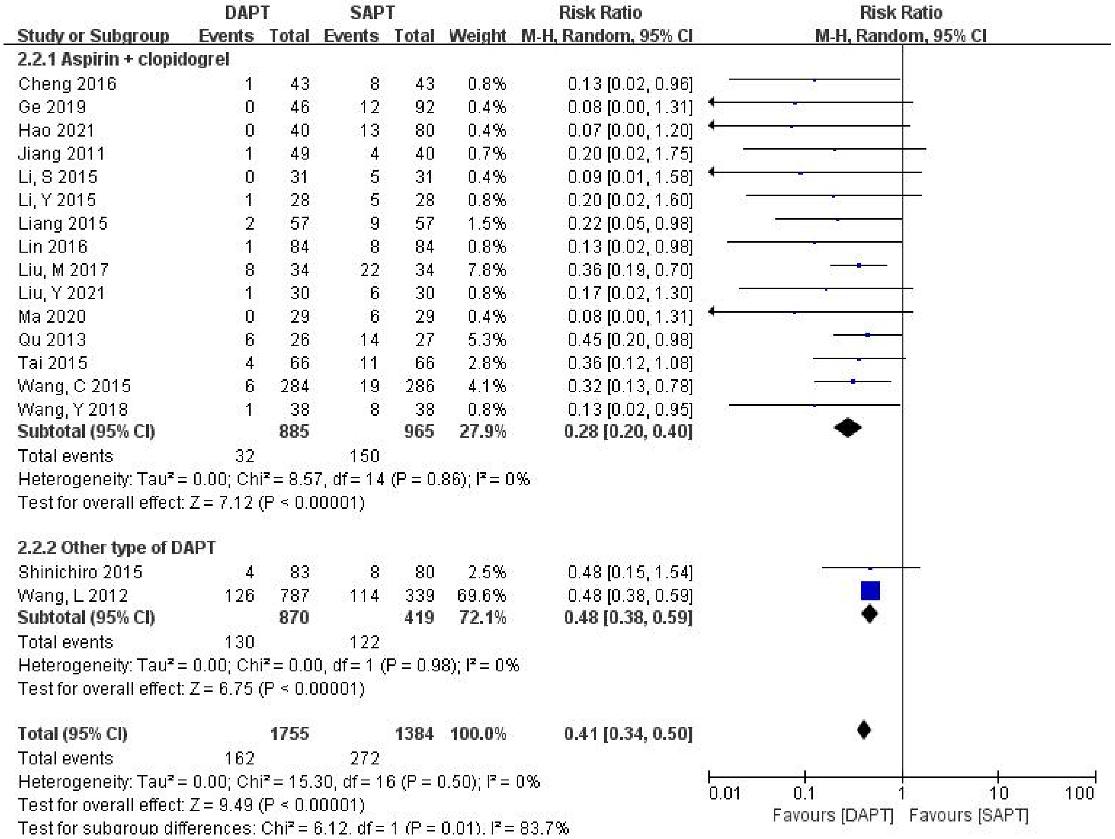

Supplemental Figure 19 Types of DAPT subgroup analysis results for the effect of DAPT compared with SAPT on recurrent stroke.

Study or Subgroup	DAPT Events	DAPT Total	SAPT Events	SAPT Total	Weight	Risk Ratio M-H, Random, 95% CI
**2.3.1 Aspirin + clopidogrel**						
Cheng 2016	1	43	8	43	1.4%	0.13 [0.02, 0.96]
Ge 2019	0	46	12	92	0.7%	0.08 [0.00, 1.31]
Hao 2021	0	40	13	80	0.7%	0.07 [0.00, 1.20]
Jiang 2011	0	49	6	40	0.7%	0.06 [0.00, 1.09]
Li, S 2015	0	31	5	31	0.7%	0.09 [0.01, 1.58]
Li, Y 2015	1	28	5	28	1.3%	0.20 [0.02, 1.60]
Liang 2015	2	57	9	57	2.5%	0.22 [0.05, 0.98]
Lin 2016	1	84	8	84	1.3%	0.13 [0.02, 0.98]
Liu, M 2017	8	34	22	34	11.0%	0.36 [0.19, 0.70]
Liu, Y 2021	1	30	6	30	1.3%	0.17 [0.02, 1.30]
Ma 2020	0	29	6	29	0.7%	0.08 [0.00, 1.31]
Qu 2013	6	26	14	27	8.0%	0.45 [0.20, 0.98]
Tai 2015	4	66	11	66	4.5%	0.36 [0.12, 1.08]
Wang, C 2015	18	284	32	286	14.3%	0.57 [0.33, 0.99]
Wang, Y 2018	1	38	8	38	1.4%	0.13 [0.02, 0.95]
**Subtotal (95% CI)**		**885**		**965**	**50.4%**	**0.34 [0.25, 0.47]**
Total events	43		165			
Heterogeneity: Tau² = 0.01; Chi² = 14.26, df = 14 (P = 0.43); I² = 2%						
Test for overall effect: Z = 6.60 (P < 0.00001)						
**2.3.2 Other type of DAPT**						
Shinichiro 2015	5	83	9	80	4.8%	0.54 [0.19, 1.53]
Wang, L 2012	168	787	152	339	44.8%	0.48 [0.40, 0.57]
**Subtotal (95% CI)**		**870**		**419**	**49.6%**	**0.48 [0.40, 0.57]**
Total events	173		161			
Heterogeneity: Tau² = 0.00; Chi² = 0.05, df = 1 (P = 0.83); I² = 0%						
Test for overall effect: Z = 8.22 (P < 0.00001)						
**Total (95% CI)**		**1755**		**1384**	**100.0%**	**0.40 [0.31, 0.51]**
Total events	216		326			
Heterogeneity: Tau² = 0.03; Chi² = 17.84, df = 16 (P = 0.33); I² = 10%						
Test for overall effect: Z = 7.53 (P < 0.00001)						
Test for subgroup differences: Chi² = 3.32, df = 1 (P = 0.07), I² = 69.9%						

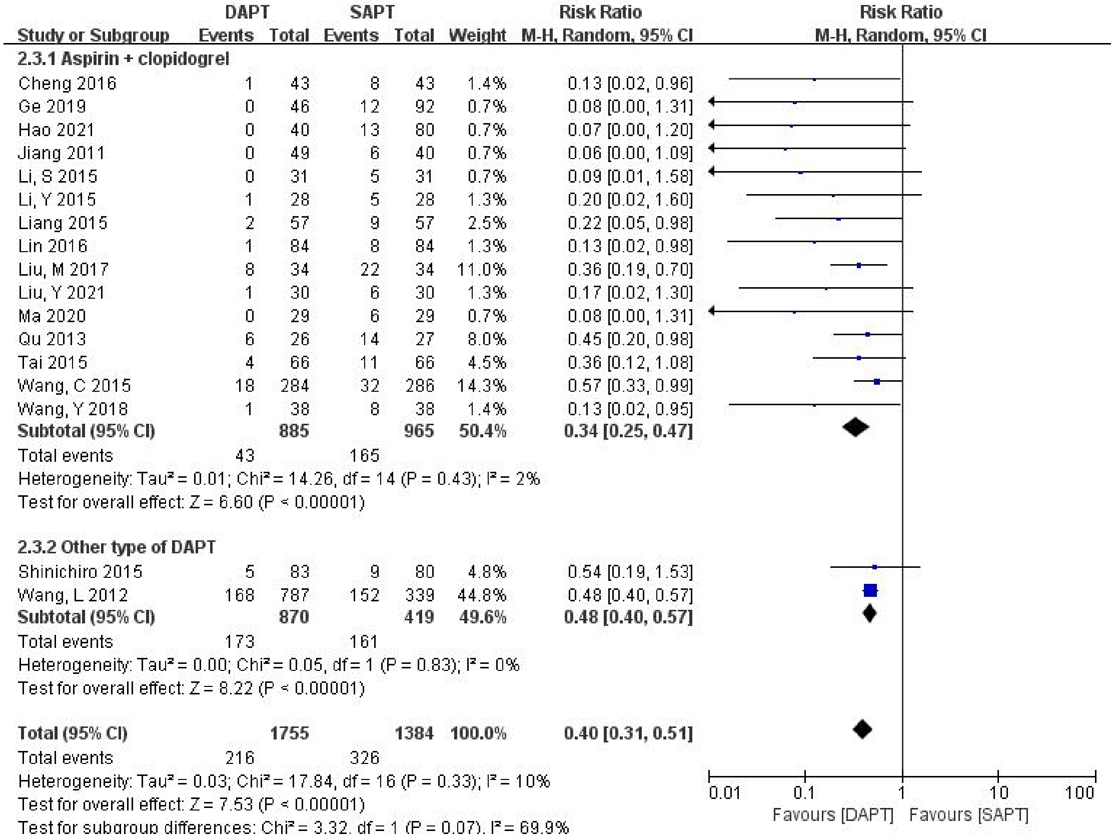

Supplemental Figure 20 Types of DAPT subgroup analysis results for the effect of DAPT compared with SAPT on ischemic events.

Study or Subgroup	DAPT Events	DAPT Total	SAPT Events	SAPT Total	Weight	Risk Ratio M-H, Random, 95% CI
**2.4.1 Aspirin + clopidogrel**						
Cheng 2016	1	43	8	43	1.1%	0.13 [0.02, 0.96]
Ge 2019	0	46	12	92	0.6%	0.08 [0.00, 1.31]
Hao 2021	0	40	13	80	0.6%	0.07 [0.00, 1.20]
Jiang 2011	0	49	3	40	0.5%	0.12 [0.01, 2.20]
Li, S 2015	0	31	5	31	0.6%	0.09 [0.01, 1.58]
Li, Y 2015	1	28	5	28	1.1%	0.20 [0.02, 1.60]
Liang 2015	2	57	9	57	2.1%	0.22 [0.05, 0.98]
Lin 2016	1	84	8	84	1.1%	0.13 [0.02, 0.98]
Liu, M 2017	8	34	22	34	10.3%	0.36 [0.19, 0.70]
Liu, Y 2021	1	30	6	30	1.1%	0.17 [0.02, 1.30]
Ma 2020	0	29	6	29	0.6%	0.08 [0.00, 1.31]
Qu 2013	6	26	14	27	7.2%	0.45 [0.20, 0.98]
Tai 2015	4	66	11	66	3.9%	0.36 [0.12, 1.08]
Wang, C 2015	5	284	18	286	4.8%	0.28 [0.11, 0.74]
Wang, Y 2018	1	38	8	38	1.1%	0.13 [0.02, 0.95]
**Subtotal (95% CI)**		**885**		**965**	**36.7%**	**0.28 [0.20, 0.40]**
Total events	30		148			
Heterogeneity: Tau² = 0.00; Chi² = 8.83, df = 14 (P = 0.84); I² = 0%						
Test for overall effect: Z = 7.12 (P < 0.00001)						
**2.4.2 Other type of DAPT**						
Shinichiro 2015	4	83	6	80	3.1%	0.64 [0.19, 2.19]
Wang, L 2012	122	787	110	339	60.3%	0.48 [0.38, 0.60]
**Subtotal (95% CI)**		**870**		**419**	**63.3%**	**0.48 [0.39, 0.60]**
Total events	126		116			
Heterogeneity: Tau² = 0.00; Chi² = 0.22, df = 1 (P = 0.64); I² = 0%						
Test for overall effect: Z = 6.48 (P < 0.00001)						
**Total (95% CI)**		**1755**		**1384**	**100.0%**	**0.39 [0.32, 0.49]**
Total events	156		264			
Heterogeneity: Tau² = 0.01; Chi² = 16.40, df = 16 (P = 0.43); I² = 2%						
Test for overall effect: Z = 8.41 (P < 0.00001)						
Test for subgroup differences: Chi² = 6.74, df = 1 (P = 0.009), I² = 85.2%						

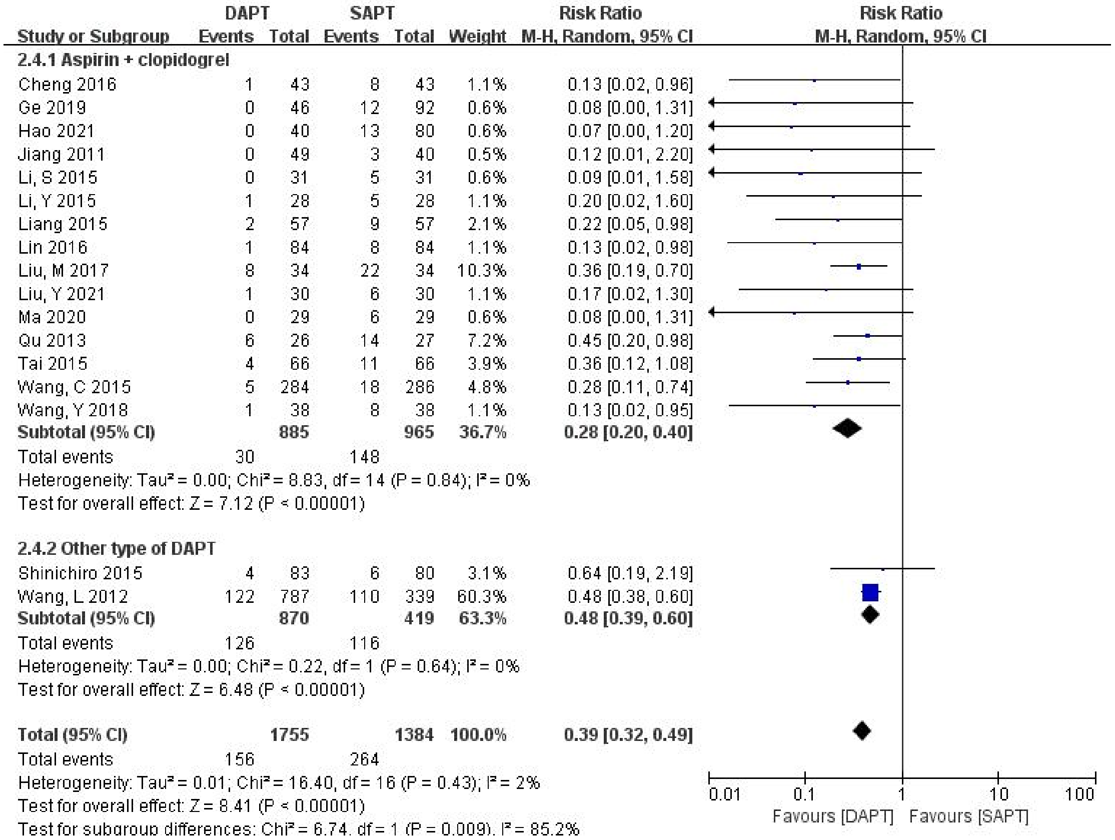

Supplemental Figure 21 Types of DAPT subgroup analysis results for the effect of DAPT compared with SAPT on recurrent cerebral infarction.

Study or Subgroup	DAPT Events	DAPT Total	SAPT Events	SAPT Total	Weight	Risk Ratio M-H, Random, 95% CI
**2.5.1 Aspirin + clopidogrel**						
Ge 2019	8	46	3	92	11.8%	5.33 [1.48, 19.16]
Hao 2021	9	40	5	80	15.8%	3.60 [1.29, 10.04]
Jiang 2011	1	49	1	40	3.3%	0.82 [0.05, 12.64]
Liu, M 2017	1	34	0	34	2.5%	3.00 [0.13, 71.15]
Liu, Y 2021	1	30	0	30	2.5%	3.00 [0.13, 70.83]
Qu 2013	0	26	0	27		Not estimable
Wang, C 2015	16	284	15	286	23.9%	1.07 [0.54, 2.13]
**Subtotal (95% CI)**		**509**		**589**	**59.9%**	**2.21 [1.09, 4.49]**
Total events	36		24			
Heterogeneity: Tau² = 0.22; Chi² = 7.18, df = 5 (P = 0.21); I² = 30%						
Test for overall effect: Z = 2.20 (P = 0.03)						
**2.5.2 Other type of DAPT**						
Shinichiro 2015	4	83	3	80	9.7%	1.29 [0.30, 5.56]
Wang, L 2012	51	787	23	339	30.4%	0.96 [0.59, 1.54]
**Subtotal (95% CI)**		**870**		**419**	**40.1%**	**0.98 [0.63, 1.54]**
Total events	55		26			
Heterogeneity: Tau² = 0.00; Chi² = 0.14, df = 1 (P = 0.71); I² = 0%						
Test for overall effect: Z = 0.08 (P = 0.94)						
**Total (95% CI)**		**1379**		**1008**	**100.0%**	**1.61 [0.96, 2.71]**
Total events	91		50			
Heterogeneity: Tau² = 0.17; Chi² = 11.03, df = 7 (P = 0.14); I² = 37%						
Test for overall effect: Z = 1.79 (P = 0.07)						
Test for subgroup differences: Chi² = 3.58, df = 1 (P = 0.06), I² = 72.0%						

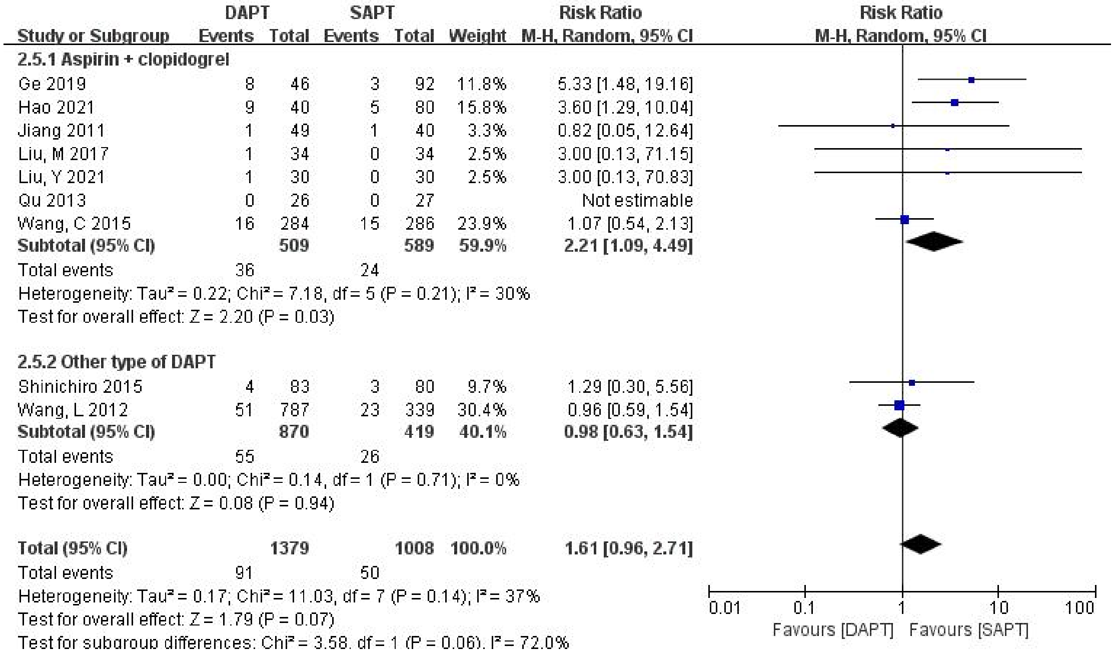

Supplemental Figure 22 Types of DAPT subgroup analysis results for the effect of

DAPT compared with SAPT on bleeding events.

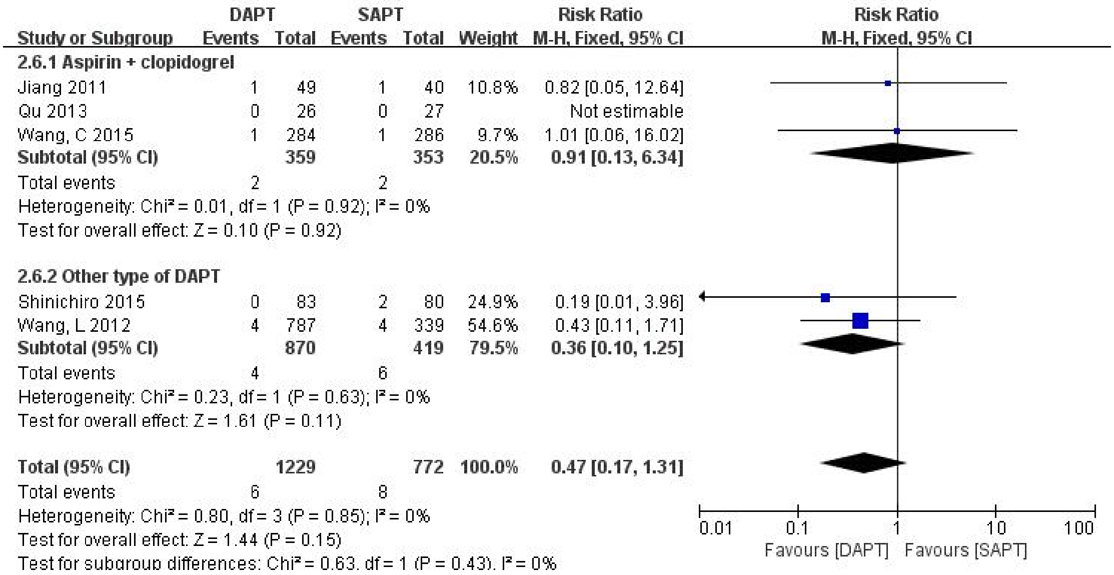

Supplemental Figure 23 Types of DAPT subgroup analysis results for the effect of DAPT compared with SAPT on cerebral hemorrhage.

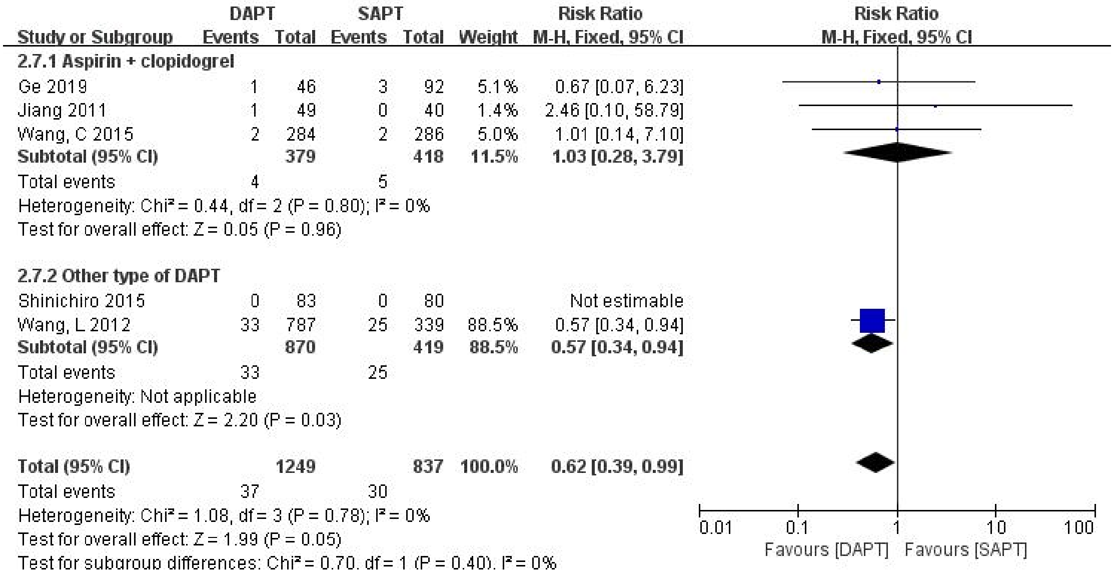

Supplemental Figure 24 Types of DAPT subgroup analysis results for the effect of DAPT compared with SAPT on death.
